# Checkpoint phosphorylation sites on budding yeast Rif1 protect nascent DNA from degradation by Sgs1-Dna2

**DOI:** 10.1371/journal.pgen.1011044

**Published:** 2023-11-13

**Authors:** Vamsi Krishna Gali, Chandre Monerawela, Yassine Laksir, Shin-ichiro Hiraga, Anne D. Donaldson

**Affiliations:** Chromosome & Cellular Dynamics Section, Institute of Medical Sciences, University of Aberdeen, Aberdeen, Scotland, United Kingdom; Columbia University, UNITED STATES

## Abstract

In budding yeast the Rif1 protein is important for protecting nascent DNA at blocked replication forks, but the mechanism has been unclear. Here we show that budding yeast Rif1 must interact with Protein Phosphatase 1 to protect nascent DNA. In the absence of Rif1, removal of either Dna2 or Sgs1 prevents nascent DNA degradation, implying that Rif1 protects nascent DNA by targeting Protein Phosphatase 1 to oppose degradation by the Sgs1-Dna2 nuclease-helicase complex. This functional role for Rif1 is conserved from yeast to human cells. Yeast Rif1 was previously identified as a target of phosphorylation by the Tel1/Mec1 checkpoint kinases, but the importance of this phosphorylation has been unclear. We find that nascent DNA protection depends on a cluster of Tel1/Mec1 consensus phosphorylation sites in the Rif1 protein sequence, indicating that the intra-S phase checkpoint acts to protect nascent DNA through Rif1 phosphorylation. Our observations uncover the pathway by which budding yeast Rif1 stabilises newly synthesised DNA, highlighting the crucial role Rif1 plays in maintaining genome stability from lower eukaryotes to humans.

## Author summary

Genome instability is a leading factor contributing to cancer. Maintaining efficient error-free replication of the genome is key to preventing genome instability. During DNA replication, replication forks can be stalled by external and intrinsic obstacles, leading to processing of nascent DNA ends to enable replication restart. However, the nascent DNA must be protected from excessive processing to prevent terminal fork arrest, which could potentially lead to more serious consequences including failure to replicate some genome sequences. Using a nascent DNA protection assay we have investigated the role of the budding yeast Rif1 protein at blocked replication forks. We find that Rif1 protects nascent DNA through a mechanism that appears conserved from yeast to humans. We show that budding yeast Rif1 protects nascent DNA by targeting Protein Phosphatase 1 activity to prevent degradation of nascent DNA by the Sgs1-Dna2 helicase-nuclease complex. Furthermore, we find that Rif1 phosphorylation by the checkpoint pathway during replication stress is crucial for this function. Our results indicate that the S phase checkpoint machinery acts by phosphorylating Rif1 to protect nascent DNA, providing important clues concerning the conserved role of Rif1 in regulating events when replication is challenged.

## Introduction

Maintaining genome integrity during replication of the genome is key to preventing oncogenesis. During S phase of the cell cycle, when DNA replication occurs, replication forks can encounter many obstacles that challenge error-free duplication of the genome. Numerous cellular proteins act to ensure the complete and accurate transmission of genomic information to daughter cells in each cell cycle.

Rif1 is one such protein, important for maintaining genome integrity at several steps of the chromosome cycle. Rif1 is a multi-functional protein conserved from budding yeast to humans, which was originally identified as a negative regulator of telomere length in the budding yeast *Saccharomyces cerevisiae* [1]. While its telomere length regulation function appears to be specific to yeast [2], other roles of Rif1 are conserved in eukaryotes [3, 4]. One apparently conserved function of Rif1 is promotion of double-stranded break (DSB) repair through non-homologous end joining (NHEJ). Rif1 drives DSB repair toward NHEJ by protecting 5’ ends from resection that would favour homology-directed repair (HDR), in a function that appears to be conserved from budding yeast to human cells [5–8]. Mammalian Rif1 also plays a role in programmed genomic rearrangements in mammalian cells, such as immunoglobulin class switching, which is a specialised form of NHEJ [5, 6].

Another conserved function of Rif1 is control of the initiation of DNA replication [9–11]. In controlling DNA replication, Rif1 acts by suppressing premature activation of the minichromosome maintenance protein (MCM) complex as the replicative helicase. In this role Rif1 operates as a substrate-targeting subunit for Protein Phosphatase 1 (PP1), directing dephosphorylation of the MCM complex, and counteracting its phosphorylation by the Dbf4-dependent kinase (DDK) to constrain replication origin activation [12–17].

Rif1 also functions at later stages of the DNA replication process. In particular, it was recently demonstrated that mammalian Rif1 protects nascent DNA at replication forks challenged by replication inhibitors [18, 19]. DNA replication forks can be impeded or stalled for many reasons. Obstacles such as collisions between the replication and transcription machinery, DNA/RNA hybrids (R-loops), ribonucleotide incorporation, DNA lesions and adducts, DNA secondary structure, repetitive DNA sequences, non-histone protein-DNA complexes, and accumulation of topological stresses may cause replication forks to stall or collapse [20, 21]. Stalled forks are frequently processed to prepare them for replication restart, with the nascent DNA subject to controlled degradation to create a single-stranded stretch that can be utilised for homology-dependent fork restart mechanisms [22]. In this context degradation can be carried out by multiple nucleases, including MRE11, EXO1, and DNA2 [23, 24]. The action of these nucleases is restricted by a number of different proteins. In mammalian cells, BRCA1 and BRCA2 protect nascent DNA from degradation by MRE11 nuclease [25], whereas BOD1L protects the nascent DNA from the DNA2-WRN nuclease-helicase complex but not from MRE11 [26]. Human Rif1 was shown to protect the nascent DNA specifically from degradation by DNA2-WRN nuclease-helicase complex, in a function that depends on Rif1 interaction with PP1. Phosphorylation of DNA2 and WRN was increased in cells depleted for Rif1, suggesting that Rif1-PP1 could potentially modulate the phosphorylation status of DNA2-WRN to control its activity [18, 19].

The proteins that process stalled replication forks are less well understood in budding yeast. While genetic studies indicate that a similar set of proteins as in human cells are important to protect cells from replication stress, their precise molecular roles remain unclear [27–30]. It was recently reported that the budding yeast MRX protein complex (composed of Mre11, Rad50, and Xrs2) promotes resection at stalled forks, but MRX appears to act in this role by supporting the remodelling of nascent chromatin, rather than through its nuclease activity [27]. Indeed the relationship of nascent DNA processing to replication fork recovery is not fully understood: some resection appears necessary to enable homology-dependent fork recovery pathways, but excessive DNA degradation is associated with genome instability [31], possibly because extensive nascent DNA loss prevents the use of the most accurate fork recovery pathways and forces cells to depend on more mutagenic pathways (reviewed by [32]). Nascent DNA degradation in the absence of mammalian RIF1 was demonstrated to be associated with genome rearrangements [19].

Throughout eukaryotes, inhibition of DNA replication causes activation of the replication, or ‘intra-S phase’, checkpoint machinery. In studying how cells respond to replication stress to maintain genome integrity, hydroxyurea (HU) has been used extensively as a model drug. HU acts by inhibiting ribonucleotide reductase leading to depletion of cellular deoxyribonucleotide triphosphate (dNTP) pools, slowing down the progression of active replication forks in the cell [33]. Inhibition of DNA synthesis generates increased single-stranded DNA as the replicative helicase proceeds uncoupled from DNA synthesis [34], causing activation of the intra-S phase checkpoint through recognition of Replication Protein A (RPA) bound to single-stranded DNA (ssDNA) [21, 35]. The Mec1 apical kinase is recruited to the RPA-coated ssDNA, and through the mediator Mrc1 activates the effector kinase Rad53 [36]. This results in a cascade of cellular responses, mediated through phosphorylation of multiple factors by Mec1 and Rad53 [35]. Activation of the intra-S phase checkpoint strongly affects the activation of further replication origins. Globally, new origin initiation events are inhibited, but in the proximity of stalled forks dormant origins are activated, at least in mammalian cells [37, 38]. At stalled replication forks the intra-S phase checkpoint is proposed to stabilise the replisome structure [39]. However, any implication of the S phase checkpoint in controlling the resection of nascent DNA at stalled forks has remained unclear.

Although the exact relationship between checkpoint activation and nascent DNA stability remains under investigation, checkpoint signalling has been implicated in stabilising nascent DNA and in modulating the protein components present at replication forks, in both mammalian cells and yeast [40, 41]. However, differences in methodologies make it difficult to precisely align results obtained from yeast with our knowledge of mammalian cell pathways. Partly to compare nascent DNA stabilisation mechanisms in yeast with those characterised for human cells, we recently deployed in *S*. *cerevisiae* a DNA combing-based nascent DNA degradation assay similar to that frequently used in mammalian cells. Using this assay we discovered that yeast Rif1 protein plays a role in protecting nascent DNA from degradation when replication is inhibited by HU [42]. The discovery aligns with the findings that mouse and human Rif1 function in nascent DNA protection [18, 19], and opens the possibility of investigating the process using yeast molecular genetic tools.

Here we examine the mechanism by which budding yeast Rif1 protects nascent DNA at stalled forks. Using a nascent DNA protection assay we find that interaction of Rif1 with PP1 (called Glc7 in yeast) is crucial to protect newly synthesized DNA during a HU block, operating to protect against Sgs1-Dna2 mediated degradation. Yeast Rif1 contains a cluster of potential or confirmed Tel1/Mec1 phosphorylation sites in its C-terminally disordered region [43–45]. We show that these sites are critical to protect nascent replicated DNA at stalled forks, indicating that the replication checkpoint machinery stimulates protection of newly-replicated DNA by phosphorylating Rif1.

## Results

### Rif1 interaction with PP1/Glc7 is crucial to protect nascent DNA from degradation under replication stress

**Fig 1:**
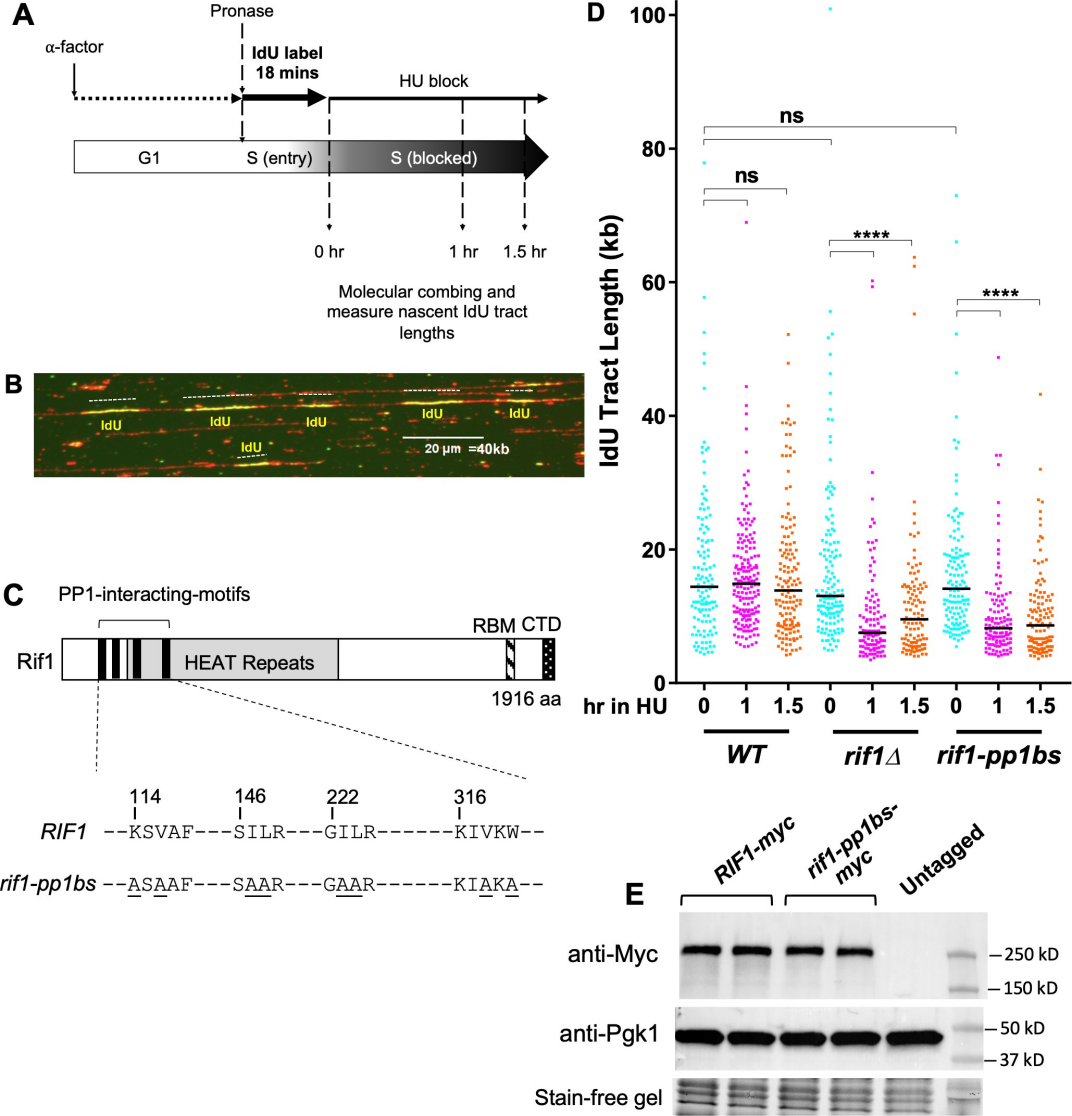
Rif1 must interact with Protein Phosphatase 1 (Glc7) to prevent degradation of nascent DNA. (A) Nascent DNA protection assay procedure. Cells arrested with α-factor were released into medium containing 1.13 mM IdU to label nascent DNA. After labelling nascent DNA for 18 mins, cells were collected by filtration and resuspended in medium containing 0.2 M HU. (B) Specimen analysis showing one fiber (stained red using anti-ssDNA) with five IdU tracts (IdU stained green). Scale bar 20 μm, equivalent to 40 kb. (C) Schematic of Rif1 showing the four PP1/Glc7-interaction motifs mutated in *rif1-pp1bs*, the series of HEAT repeats, and the Rap1-binding motif (RBM) and C-terminal domain (CTD), comprising a low-affinity Rap1 binding site and tetramerization module [8]. (D) Degradation of nascent DNA in *rif1-pp1bs* cells. Plot shows IdU tract lengths measured after incubation in a HU block for the intervals specified. ≥100 tracts were measured for each condition. In this and subsequent plots, black horizontal bars indicate median values, **** indicates *p*-values less than 0.0001 and were obtained by Mann-Whitney-Wilcoxon test, ns means “not significant”. Strains used were VGY85 (*WT*), CMY6 (*rif1Δ*) and CMY42 (*rif1-pp1bs*). (E) Western blot confirming expression of Rif1-pp1bs protein. Lanes (left to right) show duplicate *RIF1-myc* strain isolates (VGY320 and VGY321), duplicate *rif1-pp1bs-myc* isolates (VGY310 and VGY311), Untagged *RIF1* strain (YK402), and Marker. Top panel probed with anti-myc, middle panel with anti-Pgk1, and lower panel shows protein visualisation on stain-free gel. Protein samples prepared using TCA procedure.

Deletion of budding yeast Rif1 was reported to cause a defect in the protection of nascent DNA from degradation when replication fork progression is blocked by HU treatment [42]. To investigate this function of Rif1 further, we used a previously established DNA combing assay to assess the stability of labelled nascent DNA in budding yeast [42]. Briefly, cells capable of incorporating thymidine analogs were synchronized in G1 phase, then released to begin DNA replication in medium containing 5-iodo-2′-deoxyuridine (IdU). After 18 minutes, IdU was removed and HU was added, and samples collected at time points thereafter (Fig 1A). DNA from these samples was combed, and IdU-labelled nascent DNA tracts visualized by immunodetection (Fig 1B). Stability of the nascent DNA tracts during the replication block can be assessed by comparing the lengths of the nascent DNA tracts when HU is added with the tract lengths at later time points.

Association with PP1 is essential for various Rif1 functions in budding yeast and humans [13–15, 18, 46–48]. In mammalian cells nascent DNA protection by Rif1 requires PP1 interaction, so we tested if Rif1-PP1/Glc7 interaction is also needed to protect nascent DNA in budding yeast [18]. For this purpose, we used a *RIF1* allele (*rif1-pp1bs*) that has all four PP1-interacting motifs abolished (Fig 1C) by mutation of two critical residues within each motif to alanine [14]. Nascent DNA tract length labelled during the 18 min incubation did not significantly differ between wild type (*WT*), *rif1Δ* and *rif1-pp1bs* cells, revealing that the distance travelled by forks in this interval was similar in all three strains at the time of HU addition (0 hr, Fig 1A and 1D). However while nascent DNA tract length in *WT* cells did not change during the HU treatment, the median length of *rif1Δ* nascent DNA tracts was significantly decreased by 1 hr into the HU block (from 13.8 kb to 7.5 kb, Fig 1D) confirming the previous finding that Rif1 is required to prevent degradation of nascent DNA in *S. cerevisiae [42]*. In *rif1-pp1bs*, after 60 minutes in the HU block, we saw a similar decrease in the length of newly synthesized DNA tracts (from 14.5 kb to 8.2kb; Fig 1D), suggesting that Rif1 interacts with PP1 to prevent degradation of nascent DNA under replication stress in budding yeast, as in human cells.

Western blot analysis confirmed that a Myc-tagged version of the Rif1-pp1bs protein is stably expressed (Fig 1E), as previously demonstrated [14, 46]. Quantification of signals in Fig 1E indicate that Rif1-pp1bs is present at 91% of the level of the wild-type Rif1 protein. We suspect that a previous report of instability of this Rif1-pp1bs protein [49] reflects its susceptibility to degradation *in vitro* under the protein preparation conditions used in that study.

A previous study [8] reported that a mutant with amino acid substitutions K437E, K563E, and K570E in the HEAT domain (called Rif1-HOOK) is defective for DNA binding at double-strand breaks and also impairs telomere recruitment. We found however that a Rif1-HOOK mutant is competent for nascent DNA protection (S1 Fig).

### Sgs1, the yeast WRN helicase homolog, mediates nascent DNA degradation when protection by Rif1 is lacking

**Fig 2:**
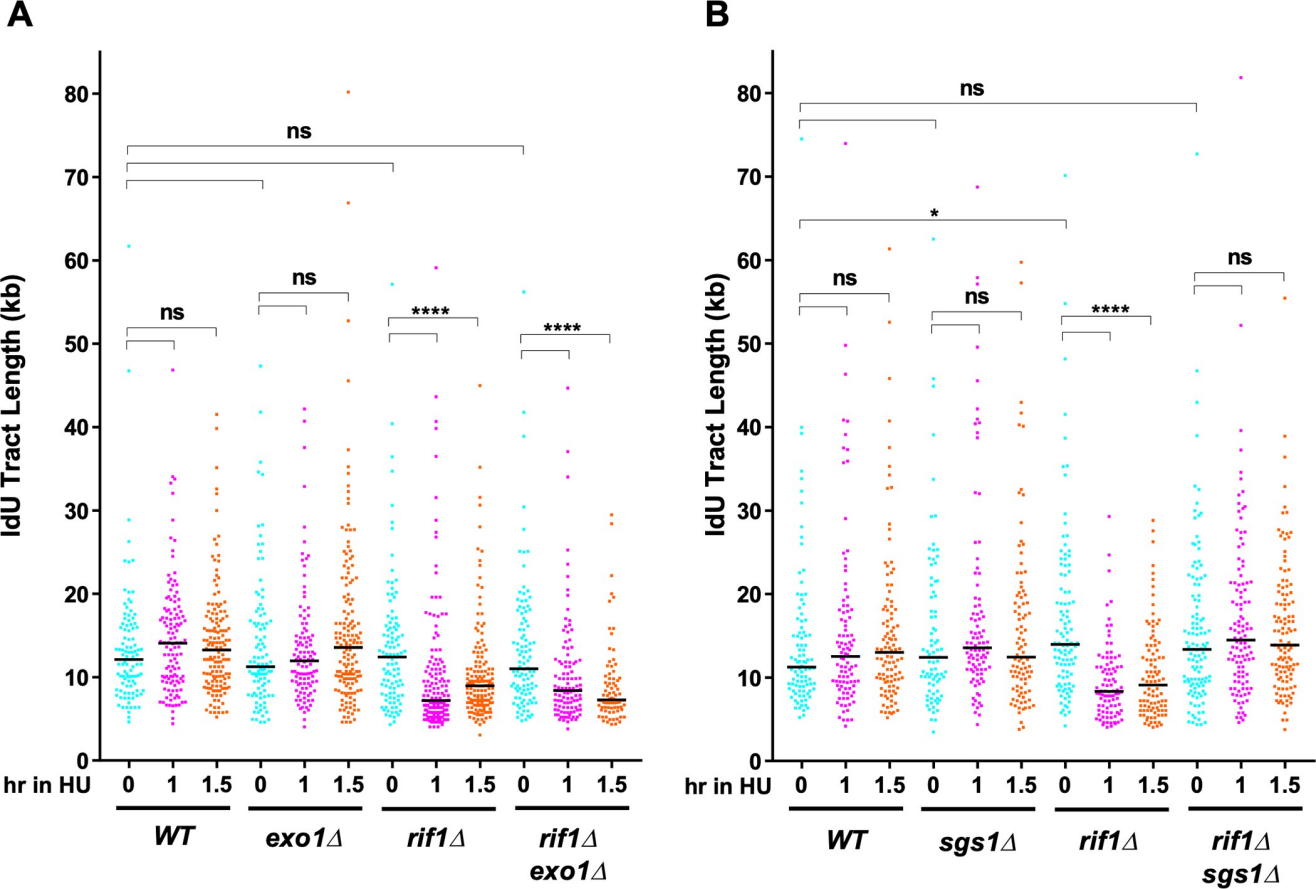
Sgs1 mediates degradation of nascent DNA in *rif1Δ* mutants. (A) *EXO1* deletion does not prevent the degradation of nascent DNA in a *rif1Δ* mutant. (B) *SGS1* deletion does prevent the degradation of nascent DNA in a *rif1Δ* mutant. IdU tracts were labelled and lengths analysed as in Fig 1. Strains used were VGY85 (*WT*), CMY6 (*rif1Δ*), CMY46 (*exo1Δ*), CMY47 (*rif1Δ exo1Δ*), CMY52 (*sgs1Δ*) and CMY53 (*rif1Δ sgs1Δ*).

In budding yeast several nucleases and helicases have been implicated in resecting nascent DNA ends [27] after remodelling by the MRX complex. Studies in mammalian cells also demonstrated that several different proteins protect nascent DNA from degradation by specific exonucleases [26, 50–53]. We tested which is the major nuclease and/or helicase responsible for nascent DNA resection in budding yeast lacking Rif1 function. We first examined the effect of deleting the 5’ to 3’ Exo1 nuclease, as in Fig 1A. If Rif1 acts by protecting the nascent DNA from degradation by Exo1, deletion of *EXO1* should rescue the nascent DNA degradation phenotype of *rif1Δ*. Removal of Exo1 did not significantly affect the initial replicated tract length after the 18 min IdU labelling period, either in the *RIF1* (*WT*) or the *rif1Δ* background (Fig 2A). After 1 hr and 1.5 hr of HU blocking, the *rif1Δ exo1Δ* mutant showed a significant decrease in IdU-labeled tract length (from 11.0 kb to 8.4kb after 1 hour HU treatment; Fig 2A), similar to the decrease of 12.4 kb to 7.2 kb observed in the *rif1Δ* single mutant. Therefore, nascent DNA degradation still occurs when Exo1 is absent, indicating that Exo1 is not the major nuclease responsible for degrading nascent DNA when Rif1 function is lacking.

We next examined whether Sgs1, the yeast homolog of the human WRN, is important for nascent DNA degradation. *sgs1Δ* and *rif1Δ sgs1Δ* strains show no significant difference in the median length of newly synthesized DNA tracts (at 0 hr) when compared to *WT* and *rif1Δ*, respectively (Fig 2B), indicating that removal of Sgs1 does not affect the progression of unblocked replication forks in the initial labelling period. However, when compared to the strong resection phenotype of *rif1Δ* cells after HU addition, *rif1Δ sgs1Δ* cells showed no significant decrease in nascent DNA tract length even after 1.5hr HU treatment (Fig 2B). Therefore, deleting Sgs1 rescues the degradation phenotype seen in a *rif1Δ* background, indicating that the budding yeast Sgs1 helicase is important for degrading nascent DNA deprotected by Rif1 removal. These observations are altogether consistent with findings in human cells lacking Rif1, where the WRN helicase was needed for nascent DNA degradation [18].

We examined the effect of HU on viability of these mutants to understand whether nascent DNA instability is directly related to HU sensitivity. Surprisingly, a *rif1Δ* mutant shows little if any HU sensitivity while an *sgs1Δ* mutant is extremely sensitive (S2 Fig). This observation might reflect the fact that at stalled forks, some processing of nascent DNA is needed to enable proper fork recovery (as in human cells [31]); but equally, could be related to other roles of Sgs1 during replication stress such as chromosome decatenation or checkpoint activation [54–56].

### Dna2 is required for the degradation of nascent DNA in cells lacking Rif1

Sgs1 acts as a helicase that unwinds dsDNA to feed an ssDNA strand to the nuclease Dna2, promoting processive resection of DNA ends [57]. Since nascent DNA degradation in the absence of yeast Rif1 requires Sgs1, we explored whether Dna2 is the major nuclease activity responsible for degradation of newly synthesized DNA in a *rif1Δ* background.

**Fig 3:**
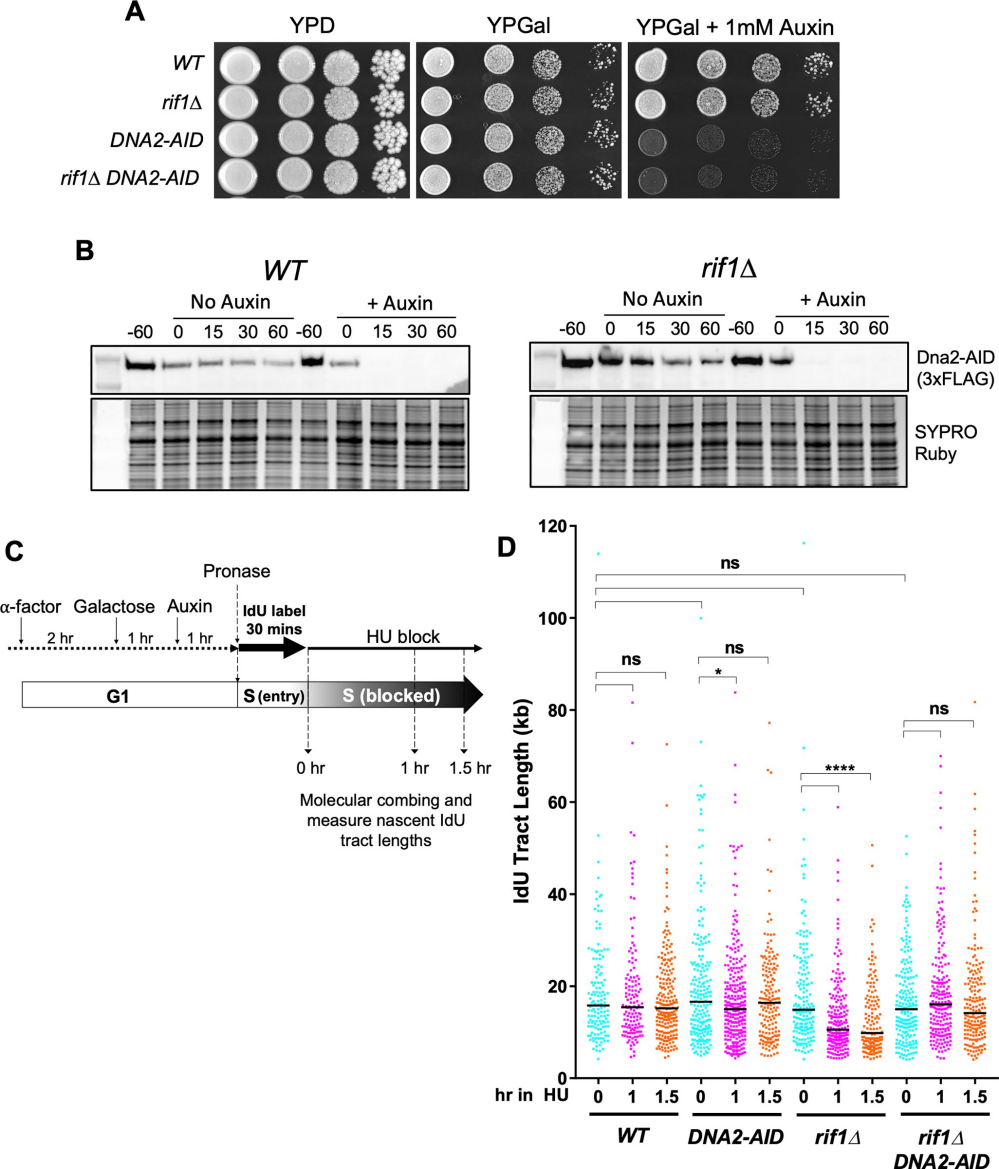
Rif1 protects nascent DNA from Dna2 mediated degradation. (A) Dna2 depletion via an AID degron tag prevents cell growth. Serial dilutions (1:10) of cells were grown on YPD, YP-Gal and YP-Gal supplemented with 1 mM auxin at 30°C. (B) Western blot using anti-FLAG antibody confirms degradation of Dna2 in wild type (left panel) and *rif1Δ* (right panel) cells. As illustrated in the schematic (below), samples were collected from G1-arrested cultures 60 mins before auxin addition (‘-60’ lanes), at the time of auxin addition (‘0’ lanes) and at timepoints thereafter. Proteins prepared by alkaline extraction method. (C) Nascent DNA protection assay procedure to test effects of Dna2 depletion, incorporating OsTIR1 induction by galactose and auxin addition to degrade Dna2. (D) Depletion of Dna2-AID prevents degradation of nascent DNA in *rif1Δ* cells. Here and below * and **** indicate *p*-values less than 0.05 and 0.0001, respectively. Strains used were CMY54 (*WT*), CMY56 (*rif1Δ*), CMY58 (*DNA2-AID*) and CMY59 (*rif1Δ DNA2-AID*).

Dna2 is an essential protein, probably because of its involvement in Okazaki fragment processing [58]. We first investigated a temperature sensitive *dna2-1* allele and the effect of combining it with *rif1Δ*, however, the strain background was unsuitable for the assay due to poor growth even at its permissive temperature [59] (S3 Fig). Therefore, we designed an auxin-inducible degradation (AID) strategy to test the involvement of Dna2 in nascent DNA degradation [60]. An AID tag was fused to the C-terminus of Dna2 in *WT* and *rif1Δ* cells, in a strain background bearing a cassette encoding OsTIR1, the E3 ubiquitin ligase that promotes degradation of AID-tagged proteins [61]. OsTIR1 is under the control of a galactose-regulated promoter, enabling induced depletion of Dna2-AID by addition of galactose and auxin. We confirmed that cells with Dna2-AID were unable to grow on plates containing galactose and auxin (Fig 3A). Western blot analysis confirmed that in α-factor-blocked cultures, Dna2-AID was swiftly degraded and became undetectable 15 min after auxin addition (Fig 3B).

To test nascent DNA protection using Dna2-AID tagged cells, we used the experimental procedure as shown in Fig 3C. Cells were first synchronized in G1 phase, then OsTIR1 was induced by addition of galactose. 1 hr later auxin was added to deplete Dna2. Replication proceeds somewhat more slowly in galactose than in glucose medium, so the initial IdU labelling period was extended to 30 min, prior to addition of HU to block replication (Fig 3C). Samples were then taken 0, 1 and 1.5 hr after HU addition for nascent DNA combing analysis.

Removal of Dna2 did not impact the initial synthesis of DNA during the 30 min IdU labelling, in either a *WT* or *rif1Δ* background (Fig 3D, compare 0 hr samples). The nascent DNA protection defect of *rif1Δ* cells was still apparent using this modified procedure (Fig 3D, *rif1Δ* cells 1 and 1.5 hr samples), with the median IdU-labelled tract length significantly decreased (from 14.9 kb to 9.8 kb) after 1.5 hours in the HU block. Depleting Dna2-AID in the *rif1Δ* background however largely prevented the degradation phenotype, with only a slight decrease in nascent DNA tract lengths over the course of the 1.5 hr HU block, which was not statistically significant (Fig 3D, *rif1Δ DNA2-AID*, 1 and 1.5 hr samples). We conclude that Dna2 is the major nuclease responsible for degradation of nascent DNA in *rif1Δ* cells. In human cells also, nascent DNA deprotected by loss of Rif1 is degraded primarily by Dna2 [18, 19]. Therefore, our results confirm that Rif1 protects nascent DNA from degradation by the Dna2-WRN/Sgs1 nuclease-helicase in yeast as well as in human cells, in the pathway that appears to be evolutionarily conserved.

### Phosphorylation of a cluster of S/TQ checkpoint recognition sites in yeast Rif1 is required for nascent DNA protection

**Fig 4:**
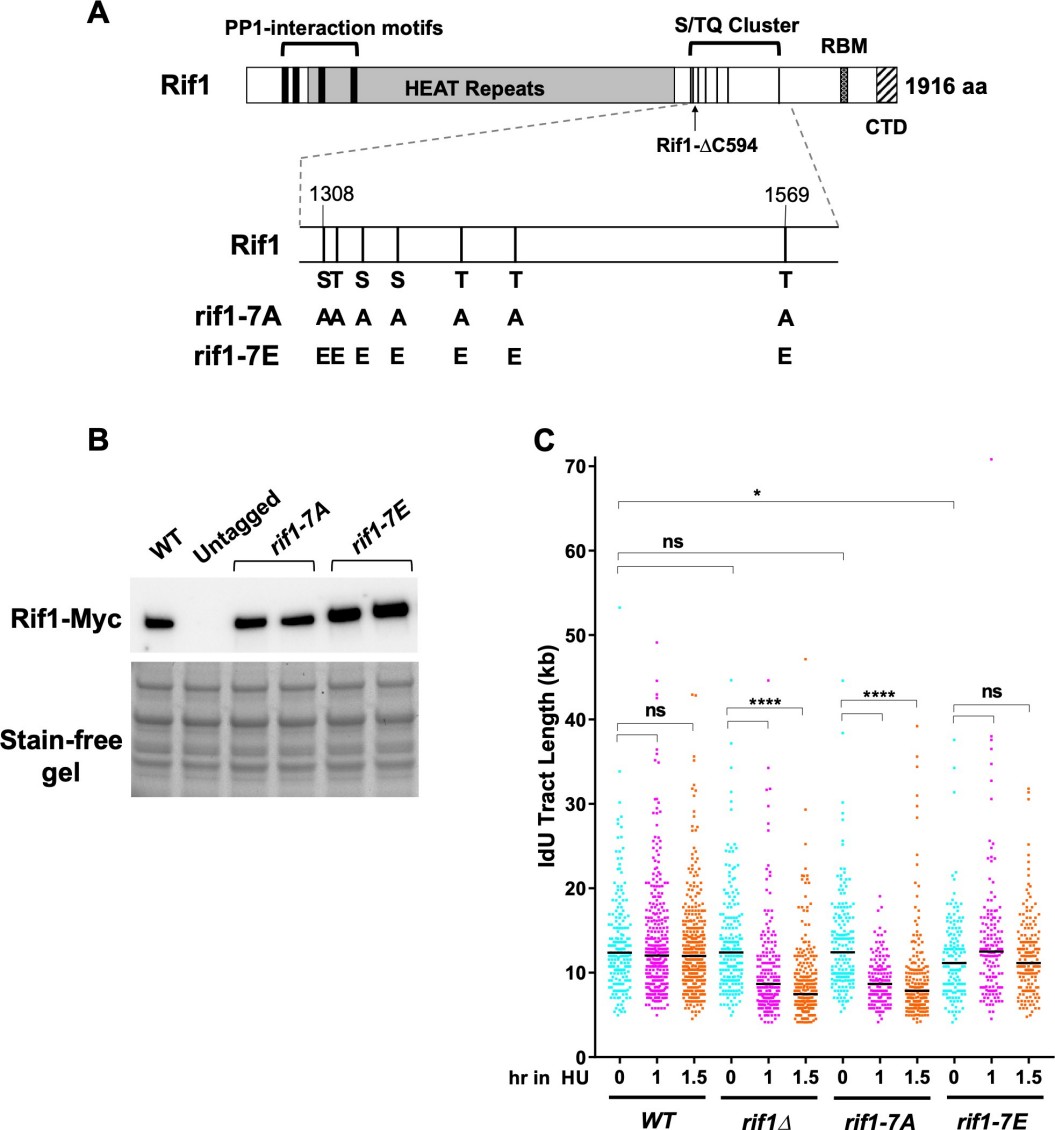
Nascent DNA protection requires a cluster of Tel1/Mec1 checkpoint recognition sites in the unstructured Rif1 C-terminal domain. (A) Schematic of Rif1 structure including seven S/TQ sites mutated to alanines in the non-phosphorylatable mutant (*rif1-7A*) or glutamic acids in the phospho-mimic mutant (*rif1-7E*). Rif1-ΔC594 mutation (1–1322) is indicated by arrow. (B) Western blot testing expression of *rif1-7A* and *rif1-7E* alleles. Strains were YSM20 (WT), YK402 (untagged), duplicate isolates CMY135 & CMY136 (*rif1-7A*), and duplicate isolates CMY137 & CMY138 (*rif1-7E*). Proteins prepared by alkaline extraction method. (C) Non-phosphorylatable mutant *rif1-7A* shows a profound defect in the protection of nascent DNA at HU-blocked forks, while nascent DNA protection is intact in the *rif1-7E* phospho-mimic mutant. Black horizontal bars indicate median values. Strains used were VGY85 (*WT*), CMY6 (*rif1Δ*), CMY130 (*rif1-7A*) and CMY132 (*rif1-7E*).

The Rif1 sequence contains a cluster of seven ‘S/TQ’ recognition motifs for the PIKK checkpoint kinases Tel1 and Mec1, located between amino acids 1308 and 1569 (Fig 4A). This region has been termed the ‘SCD’ (for ST/Q Cluster Domain) [45]. Four of these potential phosphorylation sites have been confirmed to be phosphorylated *in vivo* [44] by mass spectrometry analysis of immuno-precipitated Rif1 from yeast cells. Phosphorylation of two of these sites depends on Tel1 or Mec1 [43–45], suggesting some aspect of Rif1 function is controlled by the checkpoint machinery. However, the functional importance of these checkpoint phosphorylation sites has been unclear.

It was previously demonstrated that *rif1-ΔC594*, a C-terminal truncation mutant of Rif1, is defective for protection of nascent DNA after HU treatment [14, 42]. As indicated in Fig 4A, the Rif1-ΔC594 protein ends at amino acid 1322 and lacks five of the seven clustered consensus checkpoint phosphorylation sites in the S/TQ site cluster, as well as the C-terminal telomere interaction domain, raising the possibility that checkpoint phosphorylation contributes to nascent DNA protection by Rif1. We investigated whether phosphorylation within the S/TQ site cluster is important for Rif1 to mediate protection of newly-synthesized DNA during HU-induced replication stress. To address this issue, the serine or threonine residues at each of the seven S/TQ sites (residues 1308, 1316, 1330, 1351, 1386, 1417 and 1569) were mutated either to alanine to abolish phosphorylation (*rif1-7A*), or else to glutamic acid to mimic phosphorylation (*rif1-7E*) (Fig 4A). To confirm that *rif1-7A* and *rif1-7E* were expressed at similar levels to wild type Rif1, a 13xMyc tag was introduced at the C-termini of the mutant alleles. Western blot analysis of G1 phase-blocked cells carrying the *rif1-7A* and *rif1-7E* alleles showed similar expression levels to a strain with similarly tagged wild type Rif1 (Fig 4B).

Effects on nascent DNA protection were then tested using the procedure in Fig 1A. For the *rif1-7E* mutant, we found a slight but significant reduction in the extent of initial progression of replication forks during the initial IdU labelling period (e.g. median length 12.4 kb in wild-type versus 11.2 kb in the *rif1-7E* mutants, Fig 4C), the reason for which is unclear. With regard to nascent DNA stability after HU blockage, we found that the *rif1-7A* non-phosphorylatable allele shows a significant decrease in nascent DNA tract length (from 12.4 kb to 8.7 kb after 1 hour of HU block, Fig 4C), a reduction comparable to the decrease in nascent DNA tract length in *rif1Δ* (which in this experiment showed a reduction in tract length from 12.4 kb to 8.7 kb over the same interval, Fig 4C). A repeat of the experiment produced very similar results (S4 Fig). This defect in nascent DNA protection in *rif1-7A* suggests that phosphorylation of the S/TQ site cluster may be important for nascent DNA protection. Consistent with this idea, the phosphomimic *rif1-7E* allele in contrast showed no defect in the protection of DNA in a HU block, with the initial tract length of 11.2 kb maintained at 12.5 and 11.2 kb at 1 and 1.5 hr after HU addition (Fig 4C, also see S4 Fig). This result indicates that the *rif1-7E* allele is constitutively competent for nascent DNA protection. We conclude that, in order for Rif1 to protect nascent DNA, one or more of the clustered S/TQ checkpoint sites must be phosphorylated. Either Tel1 or Mec1 kinase is likely to be the responsible kinase. We found however that nascent DNA protection is intact in mutants lacking either Tel1 or Mec1 (S5 Fig), probably reflecting that the two kinases may be capable of substituting one another for Rif1 phosphorylation. Nonetheless, from analysis of the non-phosphorylatable and phospho-mimic mutants it appears that Rif1 checkpoint phosphorylation is important to enable nascent DNA protection after HU-induced replication stress. Despite their clear effects on nascent DNA, the *rif1-7A* and *rif1-7E* mutants showed little if any sensitivity to HU in a plate growth assay (S6 Fig), suggesting that if derailed DNA processing compromises the possibility of direct replication fork recovery after an HU block, then other pathways may be available to substitute.

### Rif1 is recruited to blocked fork independent of S/TQ checkpoint site phosphorylation

Our findings above indicate that phosphorylation of the Rif1 checkpoint site cluster is important for nascent DNA protection. One possibility is that at blocked forks, checkpoint phosphorylation of Rif1 assists with its recognition of the substrate whose dephosphorylation is important to prevent DNA degradation. An alternative possibility is that checkpoint phosphorylation is important for Rif1 recruitment to blocked forks. Recruitment of mouse RIF1 to blocked forks is at least partially dependent on its checkpoint phosphorylation [62]. We therefore tested whether the yeast Rif1-7A mutant protein shows normal recruitment to replication forks. First we used a newly constructed Rif1-V5 tagged allele to confirm previous ChIP analysis [42] showing that Rif1 binds at yeast replication origins during G1 phase (S7A Fig, G1 heatmaps). Rif1 is also present at blocked replication forks, as evidenced by broadened Rif1 ChIP signal around early-activated origins in HU, corresponding to newly initiated replication forks that have progressed some distance before stalling (S7A Fig, HU heatmaps & [42]). Subtraction of the ‘origin-bound Rif1’ (G1 phase) signal from the total signal around origins in HU-blocked cells allows estimation of the distance progressed by replication forks from origins (S7B Fig). The positions of the ‘split peaks’ visualised in this way suggest that at least some forks have progressed between 1 and 2 kb from origins under these experimental conditions (75 min after release from α factor at 23°C). Consistent with its representing Rif1 associated with blocked forks, this spreading of ChIP signal was not observed at late origins in HU-blocked cells (S7B Fig, right panel).

**Fig 5:**
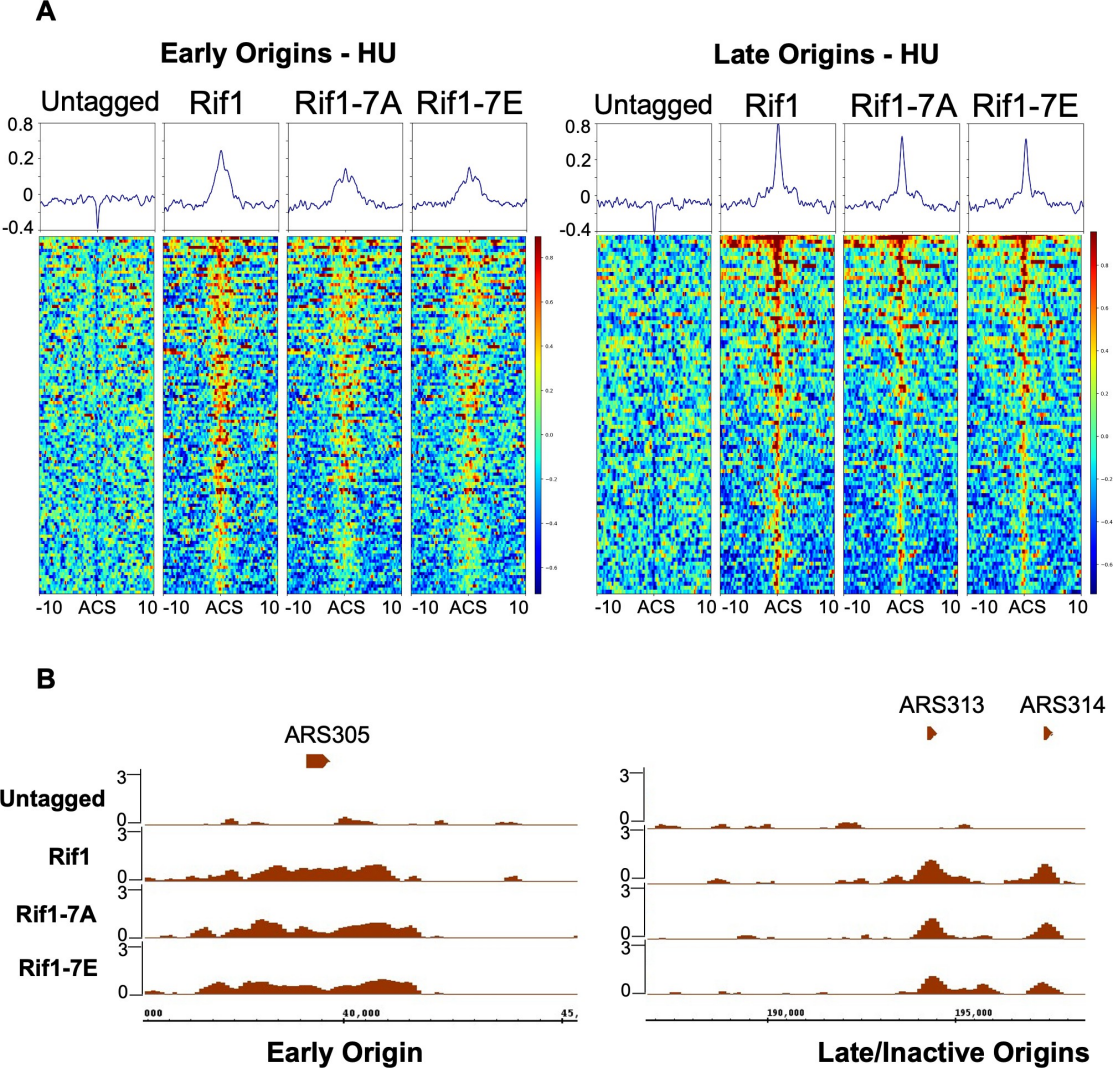
Recruitment of Rif1 to blocked forks is not dependent on phosphorylation of Rif1 at S/TQ site cluster. (A) ChIP-Seq experiment data showing enrichment of Rif1-9V5 represented as heatmaps of signal at early replication origins (left; 115 regions) or late replication origins (right; 90 regions, telomere-proximal late origins excluded). Strains were arrested in G1 phase then released into S phase in the presence of 0.2M Hydroxurea (23°C for 75 mins) before collection for ChIP-Seq experiment. After ChIP with anti-V5 antibody and normalising against Input (read count normalization) using bamcompare tool, heatmaps were generated using Plot heatmap tool on Galaxy platform. For plotting heatmaps, the centre of replication origins was used as reference point, with 10kb genomic regions added upstream and downstream for the analysis. (B) Screenshot of representative early (ARS305) and late/inactive (ARS313/ARS314) origins. Bigwig files generated as described above were visualised using Integrated Genome Browser (IGB). Strains used were YK402 (Untagged), YLY007 (*RIF1*), VGY384 (*rif1-7A*) and VGY386 (*rif1-7E*).

We next tested whether recruitment to replication forks is affected by the Rif1-7A and -7E mutations. We found that Rif1-7A is present at replication forks blocked by HU, despite the inability of this mutant protein to mediate nascent DNA protection (Fig 5A and 5B; G1 phase data for this experiment shown in S8 Fig, and additional experiment using Rif1-myc shown in S9 Fig). In some experiments Rif1-7A recruitment to blocked forks appeared slightly reduced (Fig 5A, left panel). However this mild reduction is unlikely to account for the defect of Rif1-7A in nascent DNA protection, since a similar reduction in ChIP signal was observed for Rif1-7E, which is competent for nascent DNA protection (Fig 5A, fourth pile-up from left). The phenotype of Rif1-7A indicates that phosphorylation at these seven checkpoint consensus sites is not essential for Rif1 recruitment to replication forks. Rather checkpoint phosphorylation must play a distinct role in protecting the nascent DNA, possibly by promoting recognition and interaction of Rif1-PP1 with the substrate that must be dephosphorylated to enable nascent DNA protection.

## Discussion

Here we have shown that *S*. *cerevisiae* Rif1 protects newly synthesized DNA at HU-induced stalled forks, through a process involving interaction with PP1/Glc7. We found that in the absence of Rif1 the Sgs1-Dna2 helicase-nuclease complex is primarily responsible for degrading nascent DNA (Figs 2 and 3). Our results reveal the mechanism through which Rif1 protects nascent DNA is conserved from budding yeast to humans.

Proper regulation of nascent DNA protection appears important to enable fork recovery and ensure replication stress resistance [31, 63], and the fact that the *rif1Δ* mutant does not show sensitivity to replication stress agents may reflect that other pathways are available for fork recovery. Such pathways could potentially require Sgs1, which might explain why the *sgs1Δ* mutation shows high replication stress sensitivity even though a *rif1Δ* mutant does not (S2 Fig). For example, Sgs1 has been reported to act on the rDNA during mitotic pathways of replication stress recovery, and in chromosome disentanglement [54, 55, 64].

We discovered additionally that the S/TQ phospho-site cluster located within the unstructured region of *S*. *cerevisiae* Rif1 is required for nascent DNA protection. Specifically, a non-phosphorylatable *rif1-7A* allele caused a nascent DNA protection defect comparable to that of a full *rif1Δ* deletion. A phosphomimic *rif1-7E* allele in contrast did not produce any defect, supporting the suggestion that checkpoint-mediated phosphorylation of the Rif1 S/TQ cluster is important to protect nascent DNA at stalled forks. The function of the yeast Rif1 S/TQ cluster has been the subject of debate, especially since mutating sites in this cluster does not impact telomere length in an otherwise wild-type background (although some effect on telomeres was observed in the context of *rif2* or *tel1* mutations) [45]. One site within the cluster (Rif1 S1351) was identified by a previous study as phosphorylated under replication stress conditions by Mec1 or Tel1 [43] in a proteome-wide identification of *in vivo* targets of DNA damage checkpoint kinases, confirming that the cluster of S/TQ does represent a bona fide target of Mec1/Tel1 under replication stress. These results are in alignment with effects discovered for mammalian RIF1 [62] and assign a clear physiological function for the yeast Rif1 S/TQ site cluster phosphorylation, as being important for nascent DNA protection.

Based on the effect of the *rif1-7A* and *rif1-7E* mutations, we expect that activity of either Tel1 or Mec1 will be needed for nascent DNA protection. Testing this possibility, we found that nascent DNA protection is intact in both *tel1Δ* and *sml1Δ mec1Δ* mutants (S5 Fig), probably reflecting that the two kinases can substitute for each other in phosphorylating Rif1. Removal of both Mec1 and Tel1 substantially impairs cell growth [65], and we were not able to make a conditional depletion strain suitable for testing whether nascent DNA protection is intact in the absence of both Mec1 and Tel1.

**Fig 6:**
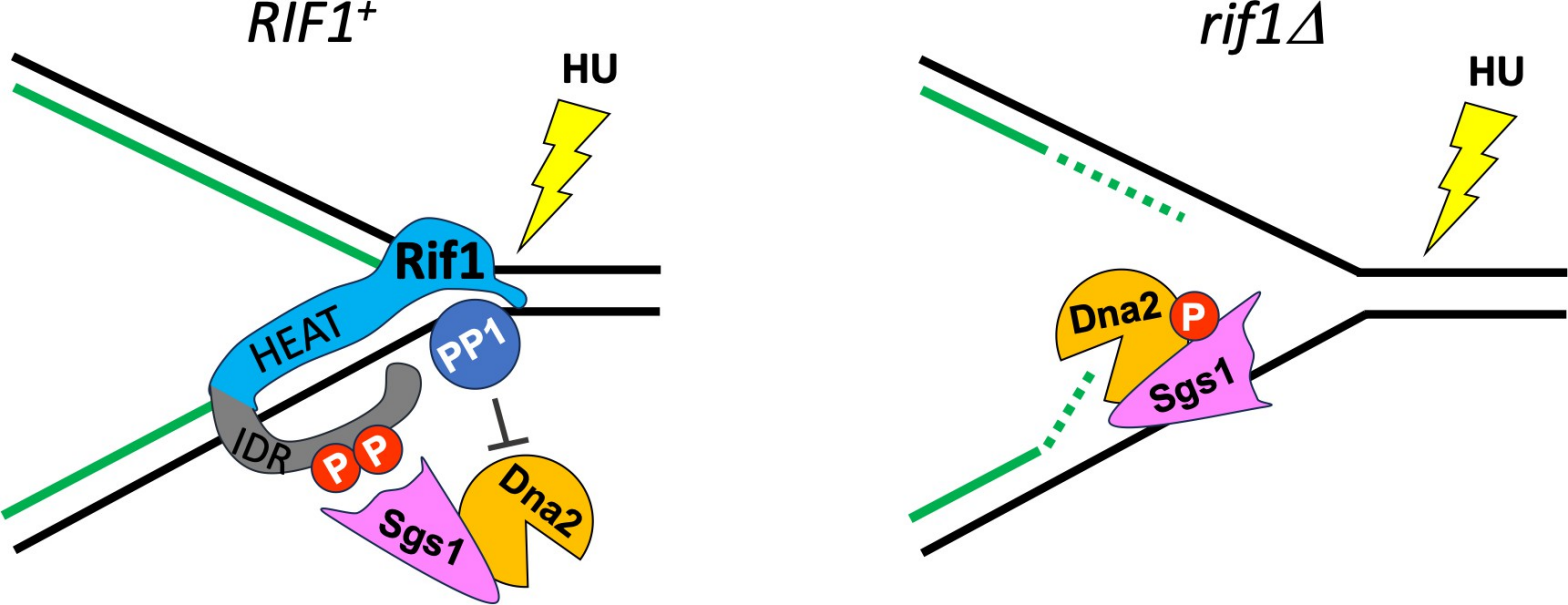
Model for protection of nascent DNA by *S*. *cerevisiae* Rif1 under HU-induced replication stress. We propose that replication fork stalling upon HU treatment causes checkpoint-mediated phosphorylation of the S/TQ site cluster in Rif1 (left panel, red circles), enabling Rif1-PP1 at the stalled replication fork to recognise the Sgs1-Dna2 complex and oppose its degradation activity, potentially by directly dephosphorylating Dna2 and/or Sgs1. In the absence of Rif1, Dna2-Sgs1 will be activated to degrade nascent DNA (right panel).

How Rif1 is recruited to stalled forks is still unclear. In human HeLa cell lines and *Drosophila*, Rif1 has been shown to interact with progressing replisomes. In *Drosophila*, fork association is largely dependent on Suppressor of Underreplication protein (SUUR) [66, 67]. Rif1 also appears to be recruited to stalled replication forks in mouse embryonic fibroblasts (MEFs) [19], and a recent study indicates that recruitment of mouse RIF1 to stalled forks in B lymphocytes depends on checkpoint phosphorylation [62]. We therefore tested whether the S/TQ site cluster is needed to stimulate the association of yeast Rif1 with replication forks upon checkpoint activation. Our results show that while it may make some minor contribution, phosphorylation of the potential Tel1/Mec1 phosphorylation site cluster in Rif1 is not essential for recruitment of yeast Rif1 to stalled forks (Fig 5), despite the fact that these sites are clearly essential for protecting the nascent DNA (Fig 4). We therefore suspect that, rather than mediating fork recruitment, phosphorylation of these sites in response to activation of the intra-S phase checkpoint pathway may be required for the proper recognition and binding to the substrate for dephosphorylation by Rif1-PP1 at replication forks (Fig 6). Phosphorylation may cause allosteric changes in Rif1 structure, in turn allowing an association with specific components recruited to stalled forks. Phosphorylation of Rif1 and of other PP1 regulatory subunits is known to modify their function by regulating PP1 interaction [68], and can plausibly be expected to modulate interaction with potential dephosphorylation targets as well.

Our results indicate that PP1 is required for Rif1 to mediate nascent DNA protection, since a *rif1-pp1bs* mutant that is incompetent for Glc7 binding cannot mediate nascent DNA protection. This protein is stably expressed in *S*. *cerevisiae*, as shown previously [14, 46] and confirmed here (Fig 1E). The target dephosphorylated by this regulation is not completely clear. The Dna2-WRN/Sgs1 complex is a good candidate, given its primary responsibility for nascent DNA degradation in both yeast and mammals lacking RIF1 (Fig 6). Previous work in mammalian cells showed that both DNA2 and WRN (which is one of five human RecQ helicase homologs with similarity to yeast Sgs1 [69]) are hyperphosphorylated in the absence of RIF1 [18, 19], suggesting that either or both DNA2 and WRN may indeed be direct targets of RIF1-PP1. MEFs treated with a PP1 inhibitor show hyperphosphorylation of DNA2 as assessed by Western blotting [19]. In Rif1-depleted human (HEK293-derived) cells, mass spectrophotometry analysis identified hyperphosphorylation of several residues in the WRN helicase either in untreated or HU-blocked conditions [18].

Our findings are consistent with the possibility that Rif1-PP1 dephosphorylates Sgs1 or Dna2 to regulate their activity. While phosphorylation of *S*. *cerevisiae* Sgs1 has been proposed to be involved in checkpoint activation through enhancing RPA and Rad53 interaction [70], any effect of Sgs1 phosphorylation on nascent DNA stability has not been investigated. In *S*. *pombe*, checkpoint-mediated phosphorylation of Dna2-S220 was proposed to enable Dna2 recruitment to replication forks and the formation and cleavage of regressed forks [41]; however, whether any equivalent phospho-site controls events at blocked forks in *S*. *cerevisiae* has not been addressed. In budding yeast, Dna2 is phosphorylated by the Cdk1 and Mec1 kinases [71]. Cdk1 phosphorylates residues T4, S17 and S237 both *in vitro* and *in vivo*, and mutating these sites to alanine leads to less processive resection of DSBs [71]. However there has been no investigation of how phosphorylation affects the activity of *S*. *cerevisiae* Dna2-Sgs1 at blocked forks. Nonetheless, since phosphorylation has been suggested to activate helicase and nuclease activities of Dna2-Sgs1, Rif1-PP1 could potentially counteract these activities by removing activating phosphorylations. A detailed, systematic study on the effect of phosphorylation on the combined Dna2-Sgs1 functional activities will need to be completed to understand how Rif1-PP1 may impact the function of this complex in nascent DNA protection.

While Sgs1 and Dna2 are good candidates, the possibility remains that Rif1-PP1 dephosphorylates other substrates to limit nascent DNA tract degradation. Various other components affect DNA protection at HU-stalled forks. For example the MRX complex acts in concert with chromatin modifiers including Set1 (catalytic component of the COMPASS complex that carries out H3K4 methylation) for remodelling of nascent chromatin to allow access by downstream helicases/nucleases to progressively resect DNA ends [27]. Various COMPASS components, such as Bre2, are potential substrates of Rif1-PP1, highlighting that Rif1 could potentially affect nascent DNA protection through COMPASS or other complexes [72].

To summarise, we have found that *S*. *cerevisiae* Rif1 protects nascent DNA by acting with PP1 to oppose the Dna2-Sgs1 helicase-nuclease complex, in a mechanism that requires Rif1 checkpoint phosphorylation and is conserved from yeast to human cells. It will be of particular interest to understand mechanistically why checkpoint phosphorylation is critical for this particular function of yeast Rif1 in protecting nascent DNA.

## Materials and methods

### Yeast strains

Yeast strains used for this study were all in a W303 *RAD5*^*+*^ background and are described in S1 Table. Plasmids and primers used in this study are listed in S2 Table and S3 Table respectively. Strains VGY85 and CMY6 were previously described [42, 73]. CMY42 was generated in a two-step process. First, a region of the N-terminus of *RIF1* (bases 97–2508) was replaced by a *URA3* cassette. This *URA3* cassette was then replaced using a PCR fragment amplified from plasmid pSH192 [14] containing mutations of the PP1 binding sites of *RIF1*. CMY46, CMY47, CMY52 and CMY53 were created by replacing the *EXO1* or *SGS1* genes with *TRP1* or *URA3* respectively, by one-step PCR replacement. To construct CMY128, first a CRISPR-Cas9 plasmid was made to enable introduction of the *dna2-1* mutation. CRISPR-Cas9 plasmid pML107 [74] was digested with BclI and SwaI restriction enzymes. The primer pair CM95-CM96, which encodes guide RNA directed towards *DNA2*, was annealed and cloned into the linearised plasmid to create plasmid CMP1. Primer CM97 was used as a ssDNA repair template to introduce the *dna2-1* mutation P504S. After transformation with CMP1 and the repair template, introduction of the correct mutation was confirmed by sequencing. *RIF1* was then replaced in CMY128 with a *HIS3* cassette, by one step PCR replacement, to create CMY134. Plasmid pMK198 (a gift from Masato Kanemaki), which contains the E3 ubiquitin ligase *OsTIR1* under the control of a *GAL* promoter, was digested with StuI and integrated into the genome of VGY85 and CMY6 at the *ura3-1* locus. The C-terminus of *DNA2* was tagged with full length AID amplified from plasmid TK12, which also included a 3xFLAG tag and nourseothricin selection marker to create CMY58 and CMY59. Integration of the AID tag was confirmed by sequencing. Rif1 phospho-site mutants were constructed by first replacing the Rif1 ORF nucleotide sequence 3903–4724 (amino acids 1302–1574) with a *URA3* cassette, removing the entire cluster of seven S/TQ sites to create a *rif1Δscd*::*URA3* strain, CMY71. From IDT Technologies, we obtained 822 bp ‘gBlock’ fragments encoding either alanine residues (7A allele) or glutamic acid residues (7E allele) instead of serine/threonine at the seven S/TQ sites. These phospho-site mutant fragments were transformed into CMY71 to replace the *URA3* cassette, creating the *rif1-7A* or *rif1-7E* strains CMY130 and CMY132, respectively. The S/TQ site mutations were confirmed by sequencing. The C-termini of the *rif1-7A* and *rif1-7E* alleles were tagged with a Myc tag by amplifying a 13xMyc-HIS3MX6 cassette from YSM20 [44] genomic DNA using primers AS85-AS86, and transforming the amplified fragment into CMY130 and CMY132. Creation of these *rif1-7A* and *rif1-7E* Myc-tagged alleles was confirmed by sequencing. For V5 tagging of Rif1 and mutant strains, YL001 and YL002 primer pair were used to amplify 9V5-KanMX4 cassette from pBH245 plasmid for transformation into required strains.

Strains that incorporate thymidine analogs were constructed by transformation with BglII restriction enzyme-digested VGP9 plasmid, to direct integration of a BrdU-Inc cassette at the *trp1-1* locus. BrdU-Inc cassette refers to 1X hENT1 and 1XHSV-TK. The *rif1-HOOK* mutant contains three mutations in Rif1(K437E K563E K570E) as described in [8]. These mutations were created by CRISPR-Cas9-based genomic modification following the method described in [74]. The VG229-VG230 primer pair (designed to encode the rif1-HOOK mutant mutations) was used to amplify a 556 bp PCR product from WT genomic DNA and used as a repair template. VGP19 plasmid which targets the HOOK domain region of Rif1 was used as guide RNA targeting plasmid. The *rif1-HOOK* mutant created was verified by sequencing.

### DNA combing

DNA combing was performed as previously described [42]. Briefly, cells were arrested with α-factor, then collected by centrifugation and resuspended in fresh media containing 1.4U /litre Pronase (to release cells into S phase) and 1.13 mM IdU (to label nascent DNA) and cultivated at 30°C. Cells were collected by filtration, washed and resuspended in fresh media containing 0.2 M HU and 5 mM thymidine. Thymidine was included to minimise labelling of ongoing DNA synthesis by any residual IdU. Cells were collected after 0, 1 and 1.5 hr and encased in low melting agarose plugs. Cells in plugs were spheroplasted and genomic DNA prepared using FiberPrep DNA extraction kit (Genomic Vision), according to manufacturer’s instructions. DNA combing was performed using FiberComb Instrument (Genomic Vision). Coverslips with combed DNA were probed with anti-IdU (Becton Dickinson 347580) and anti-ssDNA (Developmental Studies Hybridoma Bank, AB_10805144) followed by appropriate secondary antibodies with fluorescent conjugates for immunodetection. IdU tracts were visualised under a Zeiss Axio Imager.M2 microscope equipped with Zeiss MRm digital camera with a Zeiss Plan-Apochromat 63x/1.40 Oil objective lens. Images were analysed using ImageJ software. IdU-labeled tract lengths were measured using the following criteria: tracts must be at least 2 μm in length; be separated from each other by 5 μm or more; lie on a ssDNA fragment at least 50 μm in length with the tract finishing at least 5 μm from the end as visualised by ssDNA antibody. IdU tract length (in μm) was converted to kilobases using the predetermined value (2 kb/μm) for the DNA combing method.

### *dna2-1* growth plate assay

To verify temperature sensitivity of *dna2-1* mutants, strains were grown overnight in YPD. 2.5x10^5^ cells/ml were collected and serially diluted 1:5 onto YPD plates and incubated at 23°C or 30°C.

### Dna2 depletion

To investigate the effect of Dna2-AID depletion on cell viability, cells were grown overnight in YPD and 1x10^7^ cells/ml were serially diluted (1:10) the next day onto YPD, or YP+2% galactose and where required supplemented with auxin (final concentration 1 mM).

For Dna2 depletion in liquid culture using the auxin degron system, cells were grown overnight in YP+2% raffinose and arrested in G1 phase using α-factor for 2 hours. Galactose was added to a final concentration of 2% to induce expression of the E3 ubiquitin ligase OsTir1. After 1 hour, auxin (final concentration 1 mM) was added to deplete Dna2. For experiments involving labelling of nascent DNA, Dna2-depleted cells were pre-incubated with 1.13 mM IdU for 15 minutes. 1.4U /litre Pronase was then added directly (without filtration) to allow release into S phase with nascent DNA labelling. To initiate the HU block cultures were filtered and cells were resuspended in YEP 2% galactose, 1 mM auxin, 0.2 M HU and 5 mM thymidine. Samples were taken after 0, 1 and 1.5 hours and DNA combing performed as previously described [42].

### Western blotting

To confirm protein expression levels, the *RIF1* gene in WT and *rif1-pp1bs* mutant strains used for DNA combing was tagged with a 13-Myc epitope as described previously [44]. Protein extracts were prepared using the trichloroacetic acid (TCA) method based on [75]. Briefly, ~5 OD^600^ units of cells were collected and washed in 20% TCA. Cells were then resuspended in 10% TCA and disrupted using glass beads. Cell extracts were recovered, supernatant discarded and the pellet was resuspended in 200 μl of 2X sample loading buffer. Samples were boiled at 95°C for 3 min before loading on an SDS-PAGE gel for separation.

To measure Dna2-AID degradation, cells were arrested with α-factor as outlined above, then a ‘-60 min’ sample was collected. Galactose was added as above to a final concentration of 2%. 1 hr later auxin (final concentration 1 mM) was added and samples collected after 0, 15, 30 and 60 minutes. Proteins were prepared using the alkaline extraction method [76]. 175 μg and 5 μg of samples were loaded onto mini-PROTEAN 4–15% TGX gels (BIORAD) for western blotting and SYPRO staining, respectively. Dna2-AID 3xFLAG was detected using anti-FLAG M2 antibody (Sigma, F1804).

Rif1-Myc was detected using anti-Myc antibody (MBL 047–03). Loading control Pgk1 was detected using Monoclonal 459250 (Fisher Scientific).

To assess *rif1-7A* and *rif1-7E* expression levels, cells were arrested with α-factor for 2 hours and samples collected. Proteins were prepared by the alkaline extraction method [76]. 20 μg of samples were loaded onto a mini-PROTEAN 4–15% TGX Stain-Free gel (BIORAD) for western blotting.

### ChIP-Seq experiments

Chromatin Immunoprecipitation of Rif1-13Myc or Rif1-9V5 was performed as previously described [42, 77] with overnight formaldehyde cross-linking, using a polyclonal anti-Myc antibody (abcam, ab9106) for Myc epitope or V5-Tag antibody (Bio-Rad, MCA1360G). Libraries for DNA sequencing were prepared using NEBNext Ultra II DNA Library Prep Kit for Illumina (NEB, E7103S).

Bioinformatic analysis of ChIP-Seq data was performed on Galaxy platform as previously described in [42]. Briefly, Bowtie2 was used to map fastq sequencing reads to the reference genome (sacCer3). DeepTools bamCompare was used for normalising the mapped reads from IP samples to respective Input samples using readcount normalization. ChIP enrichment data obtained was then used for generating heatmaps at origins using DeepTools ComputeMatrix and DeepTools plotHeatmap.

ChIP-Seq data is uploaded to ArrayExpress under accession number E-MTAB-13451 for Rif1-13Myc datasets, and E-MTAB-13452 for Rif1-9V5 datasets.

## Supporting information

S1 Fig Rif1-HOOK mutant can mediate nascent DNA protection. Rif1 HOOK domain mutant, defective in DNA binding as described in [8], is not defective in nascent DNA protection. Black horizontal bars indicate median values. ** indicates *p*-values less than 0.01, obtained by Mann-Whitney-Wilcoxon test. ns means “not significant”. Strains used were VGY86 (*WT*), CMY6 (*rif1Δ*), VGY318 (*rif1-HOOK*).
(PDF)

S2 Fig Hydroxyurea sensitivity plate assay of *RIF1* and *SGS1* deletion mutants. Ten-fold serial dilutions of indicated strains were plated on YPD with Hydroxyurea then incubated at 30°C for 2–3 days. Strains were VGY85 (WT), CMY6 (*rif1Δ*), CMY52 (*sgs1Δ*), CMY53 (*sgs1Δ rif1Δ*).
(PDF)

S3 Fig Deletion of *RIF1* in a *dna2-1* background leads to synthetic sickness. Serial dilutions (1:5) of cells grown on YPD at 23°C and 30°C. *dna2-1* mutants are temperature sensitive and fail to grow above 30°C. Plates were imaged after 4 and 3 days respectively.
(PDF)

S4 Fig Nascent DNA protection requires a cluster of Tel1/Mec1 checkpoint recognition sites in the unstructured Rif1 C-terminal domain (repeat of experiment in Fig 4C). Analysis of the Rif1 S/TQ phospho-dead mutant *rif1-7A* reveals a defect in the protection of nascent DNA during an HU block, while fork protection is intact in the phosphomimic mutant *rif1-7E*. Black horizontal bars indicate median values. *, ** and **** indicates *p*-values less than 0.05, 0.01 and 0.0001, respectively, obtained by Mann-Whitney-Wilcoxon test. ns means “not significant”.
(PDF)

S5 Fig Deletion of *TEL1 or MEC1* does not cause a defect in nascent DNA protection. Black horizontal bars indicate median values. *, ** and **** indicates *p*-values less than 0.05, 0.01 and 0.0001, respectively, obtained by Mann-Whitney-Wilcoxon test. ns means “not significant”. Strains were (A) VGY85 (WT), CMY6 (*rif1Δ*), and VGY313 (*tel1Δ*); and (B) VGY85 (WT), CMY140 (*sml1Δ*), and CMY152 (*sml1Δ mec1Δ*).
(PDF)

S6 Fig Hydroxyurea sensitivity plate assay of *rif1-7A and rif1-7E mutants*. Serial dilutions of indicated strains were plated on YPD with no drug or with 0.1 M Hydroxyurea, then incubated at 30°C. Strains were VGY85 (WT), CMY6 (*rif1Δ*), duplicate strain isolates CMY130 & CMY131 (*rif1-7A*) and duplicate strain isolates CMY 132 & CMY133 (*rif1-7E*).
(PDF)

S7 Fig Rif1-9V5 ChIP-Seq experiment to test suitability of a V5 tag for ChIP of Rif1. (A) ChIP-Seq experiment data showing enrichment of Rif1-9V5 represented as heatmaps of signal at all replication origins (left), early-initiating origins (centre) or late-initiating replication origins (right; telomere-proximal late origins excluded). Heatmaps are shown for α factor-arrested (G1) and for HU-blocked cultures. In G1-arrested cells, Rif1 binds to both early and late origins, as previously described [42]. In HU-arrested cells, broadened distribution of Rif1-9V5 signal around early (but not late) origins is indicative of replication forks diverged from early (but not late) origins. Cells from Rif1-9V5 tagged strains were collected in either G1 phase (alpha-factor arrest) or after release into S phase in the presence of 0.2M HU (23°C, 75 min). Formaldehyde-crosslinked cells were used for IP with anti-V5 antibody. IP values normalised against Input samples were used to generate heatmaps (A) at all origins of replication (410 regions) or early origins (115 regions) or late origins (90 regions) as listed in S4 Table. (B) Heatmaps showing signal observed in HU-blocked cultures after subtraction of signal observed in G1 phase.
(PDF)

S8 Fig Heatmaps showing ChIP-Seq data from G1-arrested samples, for the experiment presented in Fig 5. ChIP-Seq experiment data showing enrichment of Rif1-9V5 represented as heatmaps of signal at early replication origins (left; 115 regions) or late replication origins (right; 90 regions, telomere-proximal late origins excluded). Strains were arrested in G1 phase before collection for ChIP-Seq analysis. Data for the same experiment for cells blocked by 0.2M Hydroxurea (23°C for 75 mins) is shown in Fig 5.
(PDF)

S9 Fig Recruitment of Rif1 to blocked forks is not dependent on phosphorylation of Rif1 at S/TQ site cluster, confirmed by analysis of Rif1-13Myc. Data from ChIP-Seq experiment using Rif1-13Myc tag, confirming analysis in Fig 5 showing that Rif1-7A mutant is not defective in recruitment to blocked replication forks. Experimental procedure was the same as that described for Figs 5 and, but using Rif1-13Myc instead of Rif1-9V5. Strains were YSM20 (*RIF1*), VGY333 (*rif1-7A*), VGY335 (*rif1-7E*).
(PDF)

S1 Table Yeast strains used in this study. (XLSX)

S2 Table Plasmids used in this study. (XLSX)

S3 Table Primers used in this study. (XLSX)

S4 Table List of Origins used for ChIP-Seq data analysis. (XLSX)

## References

[1] Hardy CF, Sussel L, Shore D. A RAP1-interacting protein involved in transcriptional silencing and telomere length regulation. Genes Dev. 1992;6(5):801–14. doi: 10.1101/gad.6.5.801 . 1577274

[2] Mattarocci S, Hafner L, Lezaja A, Shyian M, Shore D. Rif1: A Conserved Regulator of DNA Replication and Repair Hijacked by Telomeres in Yeasts. Front Genet. 2016;7:45. Epub 20160330. doi: 10.3389/fgene.2016.00045 ; PubMed Central PMCID: PMC4811881. 27066066 PMC4811881

[3] Fontana GA, Reinert JK, Thoma NH, Rass U. Shepherding DNA ends: Rif1 protects telomeres and chromosome breaks. Microb Cell. 2018;5(7):327–43. Epub 20180517. doi: 10.15698/mic2018.07.639 ; PubMed Central PMCID: PMC6035837. 29992129 PMC6035837

[4] Richards L, Das S, Nordman JT. Rif1-Dependent Control of Replication Timing. Genes (Basel). 2022;13(3). Epub 20220320. doi: 10.3390/genes13030550 ; PubMed Central PMCID: PMC8955891. 35328102 PMC8955891

[5] Chapman JR, Barral P, Vannier JB, Borel V, Steger M, Tomas-Loba A, et al. RIF1 is essential for 53BP1-dependent nonhomologous end joining and suppression of DNA double-strand break resection. Mol Cell. 2013;49(5):858–71. Epub 20130117. doi: 10.1016/j.molcel.2013.01.002 ; PubMed Central PMCID: PMC3594748. 23333305 PMC3594748

[6] Escribano-Diaz C, Orthwein A, Fradet-Turcotte A, Xing M, Young JT, Tkac J, et al. A cell cycle-dependent regulatory circuit composed of 53BP1-RIF1 and BRCA1-CtIP controls DNA repair pathway choice. Mol Cell. 2013;49(5):872–83. Epub 20130117. doi: 10.1016/j.molcel.2013.01.001 . 23333306

[7] Fontana GA, Hess D, Reinert JK, Mattarocci S, Falquet B, Klein D, et al. Rif1 S-acylation mediates DNA double-strand break repair at the inner nuclear membrane. Nat Commun. 2019;10(1):2535. Epub 20190610. doi: 10.1038/s41467-019-10349-z ; PubMed Central PMCID: PMC6557901. 31182712 PMC6557901

[8] Mattarocci S, Reinert JK, Bunker RD, Fontana GA, Shi T, Klein D, et al. Rif1 maintains telomeres and mediates DNA repair by encasing DNA ends. Nat Struct Mol Biol. 2017;24(7):588–95. Epub 20170612. doi: 10.1038/nsmb.3420 . 28604726

[9] Cornacchia D, Dileep V, Quivy JP, Foti R, Tili F, Santarella-Mellwig R, et al. Mouse Rif1 is a key regulator of the replication-timing programme in mammalian cells. EMBO J. 2012;31(18):3678–90. Epub 20120731. doi: 10.1038/emboj.2012.214 ; PubMed Central PMCID: PMC3442270. 22850673 PMC3442270

[10] Hayano M, Kanoh Y, Matsumoto S, Renard-Guillet C, Shirahige K, Masai H. Rif1 is a global regulator of timing of replication origin firing in fission yeast. Genes Dev. 2012;26(2):137–50. doi: 10.1101/gad.178491.111 ; PubMed Central PMCID: PMC3273838. 22279046 PMC3273838

[11] Yamazaki S, Ishii A, Kanoh Y, Oda M, Nishito Y, Masai H. Rif1 regulates the replication timing domains on the human genome. EMBO J. 2012;31(18):3667–77. Epub 20120731. doi: 10.1038/emboj.2012.180 ; PubMed Central PMCID: PMC3442267. 22850674 PMC3442267

[12] Alver RC, Chadha GS, Gillespie PJ, Blow JJ. Reversal of DDK-Mediated MCM Phosphorylation by Rif1-PP1 Regulates Replication Initiation and Replisome Stability Independently of ATR/Chk1. Cell Rep. 2017;18(10):2508–20. doi: 10.1016/j.celrep.2017.02.042 ; PubMed Central PMCID: PMC5357733. 28273463 PMC5357733

[13] Dave A, Cooley C, Garg M, Bianchi A. Protein phosphatase 1 recruitment by Rif1 regulates DNA replication origin firing by counteracting DDK activity. Cell Rep. 2014;7(1):53–61. Epub 20140320. doi: 10.1016/j.celrep.2014.02.019 ; PubMed Central PMCID: PMC3989773. 24656819 PMC3989773

[14] Hiraga S, Alvino GM, Chang F, Lian HY, Sridhar A, Kubota T, et al. Rif1 controls DNA replication by directing Protein Phosphatase 1 to reverse Cdc7-mediated phosphorylation of the MCM complex. Genes Dev. 2014;28(4):372–83. doi: 10.1101/gad.231258.113 ; PubMed Central PMCID: PMC3937515. 24532715 PMC3937515

[15] Hiraga SI, Ly T, Garzon J, Horejsi Z, Ohkubo YN, Endo A, et al. Human RIF1 and protein phosphatase 1 stimulate DNA replication origin licensing but suppress origin activation. EMBO Rep. 2017;18(3):403–19. Epub 20170111. doi: 10.15252/embr.201641983 ; PubMed Central PMCID: PMC5331243. 28077461 PMC5331243

[16] Mattarocci S, Shyian M, Lemmens L, Damay P, Altintas DM, Shi T, et al. Rif1 controls DNA replication timing in yeast through the PP1 phosphatase Glc7. Cell Rep. 2014;7(1):62–9. Epub 20140327. doi: 10.1016/j.celrep.2014.03.010 . 24685139

[17] Sukackaite R, Cornacchia D, Jensen MR, Mas PJ, Blackledge M, Enervald E, et al. Mouse Rif1 is a regulatory subunit of protein phosphatase 1 (PP1). Sci Rep. 2017;7(1):2119. Epub 20170518. doi: 10.1038/s41598-017-01910-1 ; PubMed Central PMCID: PMC5437018. 28522851 PMC5437018

[18] Garzon J, Ursich S, Lopes M, Hiraga SI, Donaldson AD. Human RIF1-Protein Phosphatase 1 Prevents Degradation and Breakage of Nascent DNA on Replication Stalling. Cell Rep. 2019;27(9):2558–66 e4. doi: 10.1016/j.celrep.2019.05.002 ; PubMed Central PMCID: PMC6547018. 31141682 PMC6547018

[19] Mukherjee C, Tripathi V, Manolika EM, Heijink AM, Ricci G, Merzouk S, et al. RIF1 promotes replication fork protection and efficient restart to maintain genome stability. Nat Commun. 2019;10(1):3287. Epub 20190723. doi: 10.1038/s41467-019-11246-1 ; PubMed Central PMCID: PMC6650494. 31337767 PMC6650494

[20] Mirkin EV, Mirkin SM. Replication fork stalling at natural impediments. Microbiol Mol Biol Rev. 2007;71(1):13–35. doi: 10.1128/MMBR.00030-06 ; PubMed Central PMCID: PMC1847372. 17347517 PMC1847372

[21] Zeman MK, Cimprich KA. Causes and consequences of replication stress. Nat Cell Biol. 2014;16(1):2–9. doi: 10.1038/ncb2897 ; PubMed Central PMCID: PMC4354890. 24366029 PMC4354890

[22] Ait Saada A, Lambert SAE, Carr AM. Preserving replication fork integrity and competence via the homologous recombination pathway. DNA Repair (Amst). 2018;71:135–47. Epub 20180825. doi: 10.1016/j.dnarep.2018.08.017 ; PubMed Central PMCID: PMC6219450. 30220600 PMC6219450

[23] Berti M, Vindigni A. Replication stress: getting back on track. Nat Struct Mol Biol. 2016;23(2):103–9. doi: 10.1038/nsmb.3163 ; PubMed Central PMCID: PMC5125612. 26840898 PMC5125612

[24] Neelsen KJ, Lopes M. Replication fork reversal in eukaryotes: from dead end to dynamic response. Nat Rev Mol Cell Biol. 2015;16(4):207–20. Epub 20150225. doi: 10.1038/nrm3935 . 25714681

[25] Ray Chaudhuri A, Callen E, Ding X, Gogola E, Duarte AA, Lee JE, et al. Replication fork stability confers chemoresistance in BRCA-deficient cells. Nature. 2016;535(7612):382–7. doi: 10.1038/nature18325 ; PubMed Central PMCID: PMC4959813. 27443740 PMC4959813

[26] Higgs MR, Reynolds JJ, Winczura A, Blackford AN, Borel V, Miller ES, et al. BOD1L Is Required to Suppress Deleterious Resection of Stressed Replication Forks. Mol Cell. 2015;59(3):462–77. Epub 20150709. doi: 10.1016/j.molcel.2015.06.007 . 26166705

[27] Delamarre A, Barthe A, de la Roche Saint-Andre C, Luciano P, Forey R, Padioleau I, et al. MRX Increases Chromatin Accessibility at Stalled Replication Forks to Promote Nascent DNA Resection and Cohesin Loading. Mol Cell. 2020;77(2):395–410 e3. Epub 20191120. doi: 10.1016/j.molcel.2019.10.029 . 31759824

[28] Martina M, Bonetti D, Villa M, Lucchini G, Longhese MP. Saccharomyces cerevisiae Rif1 cooperates with MRX-Sae2 in promoting DNA-end resection. EMBO Rep. 2014;15(6):695–704. Epub 20140401. doi: 10.1002/embr.201338338 ; PubMed Central PMCID: PMC4197880. 24692507 PMC4197880

[29] Villa M, Bonetti D, Carraro M, Longhese MP. Rad9/53BP1 protects stalled replication forks from degradation in Mec1/ATR-defective cells. EMBO Rep. 2018;19(2):351–67. Epub 20180104. doi: 10.15252/embr.201744910 ; PubMed Central PMCID: PMC5797966. 29301856 PMC5797966

[30] Zhu Z, Chung WH, Shim EY, Lee SE, Ira G. Sgs1 helicase and two nucleases Dna2 and Exo1 resect DNA double-strand break ends. Cell. 2008;134(6):981–94. doi: 10.1016/j.cell.2008.08.037 ; PubMed Central PMCID: PMC2662516. 18805091 PMC2662516

[31] Thangavel S, Berti M, Levikova M, Pinto C, Gomathinayagam S, Vujanovic M, et al. DNA2 drives processing and restart of reversed replication forks in human cells. J Cell Biol. 2015;208(5):545–62. doi: 10.1083/jcb.201406100 ; PubMed Central PMCID: PMC4347643. 25733713 PMC4347643

[32] Tye S, Ronson GE, Morris JR. A fork in the road: Where homologous recombination and stalled replication fork protection part ways. Semin Cell Dev Biol. 2021;113:14–26. Epub 20200709. doi: 10.1016/j.semcdb.2020.07.004 ; PubMed Central PMCID: PMC8082280. 32653304 PMC8082280

[33] Poli J, Tsaponina O, Crabbe L, Keszthelyi A, Pantesco V, Chabes A, et al. dNTP pools determine fork progression and origin usage under replication stress. EMBO J. 2012;31(4):883–94. Epub 20120110. doi: 10.1038/emboj.2011.470 ; PubMed Central PMCID: PMC3280562. 22234185 PMC3280562

[34] Taylor MRG, Yeeles JTP. Dynamics of Replication Fork Progression Following Helicase-Polymerase Uncoupling in Eukaryotes. J Mol Biol. 2019;431(10):2040–9. Epub 20190317. doi: 10.1016/j.jmb.2019.03.011 ; PubMed Central PMCID: PMC6525111. 30894292 PMC6525111

[35] Branzei D, Foiani M. Maintaining genome stability at the replication fork. Nat Rev Mol Cell Biol. 2010;11(3):208–19. doi: 10.1038/nrm2852 . 20177396

[36] Pardo B, Crabbe L, Pasero P. Signaling pathways of replication stress in yeast. FEMS Yeast Res. 2017;17(2). doi: 10.1093/femsyr/fow101 . 27915243

[37] Ge XQ, Jackson DA, Blow JJ. Dormant origins licensed by excess Mcm2-7 are required for human cells to survive replicative stress. Genes Dev. 2007;21(24):3331–41. doi: 10.1101/gad.457807 ; PubMed Central PMCID: PMC2113033. 18079179 PMC2113033

[38] Yekezare M, Gomez-Gonzalez B, Diffley JF. Controlling DNA replication origins in response to DNA damage—inhibit globally, activate locally. J Cell Sci. 2013;126(Pt 6):1297–306. doi: 10.1242/jcs.096701 . 23645160

[39] Segurado M, Tercero JA. The S-phase checkpoint: targeting the replication fork. Biol Cell. 2009;101(11):617–27. Epub 20090819. doi: 10.1042/BC20090053 . 19686094

[40] Dungrawala H, Rose KL, Bhat KP, Mohni KN, Glick GG, Couch FB, et al. The Replication Checkpoint Prevents Two Types of Fork Collapse without Regulating Replisome Stability. Mol Cell. 2015;59(6):998–1010. Epub 20150910. doi: 10.1016/j.molcel.2015.07.030 ; PubMed Central PMCID: PMC4575883. 26365379 PMC4575883

[41] Hu J, Sun L, Shen F, Chen Y, Hua Y, Liu Y, et al. The intra-S phase checkpoint targets Dna2 to prevent stalled replication forks from reversing. Cell. 2012;149(6):1221–32. doi: 10.1016/j.cell.2012.04.030 . 22682245

[42] Hiraga SI, Monerawela C, Katou Y, Shaw S, Clark KR, Shirahige K, et al. Budding yeast Rif1 binds to replication origins and protects DNA at blocked replication forks. EMBO Rep. 2018;19(9). Epub 20180813. doi: 10.15252/embr.201846222 ; PubMed Central PMCID: PMC6123642. 30104203 PMC6123642

[43] Smolka MB, Albuquerque CP, Chen SH, Zhou H. Proteome-wide identification of in vivo targets of DNA damage checkpoint kinases. Proc Natl Acad Sci U S A. 2007;104(25):10364–9. Epub 20070611. doi: 10.1073/pnas.0701622104 ; PubMed Central PMCID: PMC1965519. 17563356 PMC1965519

[44] Sridhar A, Kedziora S, Donaldson AD. At short telomeres Tel1 directs early replication and phosphorylates Rif1. PLoS Genet. 2014;10(10):e1004691. Epub 20141016. doi: 10.1371/journal.pgen.1004691 ; PubMed Central PMCID: PMC4199499. 25329891 PMC4199499

[45] Wang J, Zhang H, Al Shibar M, Willard B, Ray A, Runge KW. Rif1 phosphorylation site analysis in telomere length regulation and the response to damaged telomeres. DNA Repair (Amst). 2018;65:26–33. Epub 20180307. doi: 10.1016/j.dnarep.2018.03.001 ; PubMed Central PMCID: PMC5911405. 29544213 PMC5911405

[46] Kedziora S, Gali VK, Wilson RHC, Clark KRM, Nieduszynski CA, Hiraga SI, et al. Rif1 acts through Protein Phosphatase 1 but independent of replication timing to suppress telomere extension in budding yeast. Nucleic Acids Res. 2018;46(8):3993–4003. doi: 10.1093/nar/gky132 ; PubMed Central PMCID: PMC5934629. 29529242 PMC5934629

[47] Isobe SY, Hiraga SI, Nagao K, Sasanuma H, Donaldson AD, Obuse C. Protein phosphatase 1 acts as a RIF1 effector to suppress DSB resection prior to Shieldin action. Cell Rep. 2021;36(2):109383. doi: 10.1016/j.celrep.2021.109383 ; PubMed Central PMCID: PMC8293623. 34260925 PMC8293623

[48] Gnan S, Flyamer IM, Klein KN, Castelli E, Rapp A, Maiser A, et al. Nuclear organisation and replication timing are coupled through RIF1-PP1 interaction. Nat Commun. 2021;12(1):2910. Epub 20210518. doi: 10.1038/s41467-021-22899-2 ; PubMed Central PMCID: PMC8131703. 34006872 PMC8131703

[49] Shubin CB, Greider CW. The role of Rif1 in telomere length regulation is separable from its role in origin firing. Elife. 2020;9. Epub 2020/07/01. doi: 10.7554/eLife.58066 ; PubMed Central PMCID: PMC7371424. 32597753 PMC7371424

[50] Hashimoto Y, Ray Chaudhuri A, Lopes M, Costanzo V. Rad51 protects nascent DNA from Mre11-dependent degradation and promotes continuous DNA synthesis. Nat Struct Mol Biol. 2010;17(11):1305–11. Epub 20101010. doi: 10.1038/nsmb.1927 ; PubMed Central PMCID: PMC4306207. 20935632 PMC4306207

[51] Przetocka S, Porro A, Bolck HA, Walker C, Lezaja A, Trenner A, et al. CtIP-Mediated Fork Protection Synergizes with BRCA1 to Suppress Genomic Instability upon DNA Replication Stress. Mol Cell. 2018;72(3):568–82 e6. Epub 20181018. doi: 10.1016/j.molcel.2018.09.014 . 30344097

[52] Schlacher K, Christ N, Siaud N, Egashira A, Wu H, Jasin M. Double-strand break repair-independent role for BRCA2 in blocking stalled replication fork degradation by MRE11. Cell. 2011;145(4):529–42. doi: 10.1016/j.cell.2011.03.041 ; PubMed Central PMCID: PMC3261725. 21565612 PMC3261725

[53] Xu S, Wu X, Wu L, Castillo A, Liu J, Atkinson E, et al. Abro1 maintains genome stability and limits replication stress by protecting replication fork stability. Genes Dev. 2017;31(14):1469–82. doi: 10.1101/gad.299172.117 ; PubMed Central PMCID: PMC5588928. 28860160 PMC5588928

[54] Cejka P, Plank JL, Dombrowski CC, Kowalczykowski SC. Decatenation of DNA by the S. cerevisiae Sgs1-Top3-Rmi1 and RPA complex: a mechanism for disentangling chromosomes. Mol Cell. 2012;47(6):886–96. Epub 2012/08/14. doi: 10.1016/j.molcel.2012.06.032 ; PubMed Central PMCID: PMC3462259. 22885009 PMC3462259

[55] Bjergbaek L, Cobb JA, Tsai-Pflugfelder M, Gasser SM. Mechanistically distinct roles for Sgs1p in checkpoint activation and replication fork maintenance. EMBO J. 2005;24(2):405–17. Epub 2004/12/24. doi: 10.1038/sj.emboj.7600511 ; PubMed Central PMCID: PMC545806. 15616582 PMC545806

[56] Balogun FO, Truman AW, Kron SJ. DNA resection proteins Sgs1 and Exo1 are required for G1 checkpoint activation in budding yeast. DNA Repair (Amst). 2013;12(9):751–60. Epub 2013/07/10. doi: 10.1016/j.dnarep.2013.06.003 ; PubMed Central PMCID: PMC3769955. 23835406 PMC3769955

[57] Cejka P, Cannavo E, Polaczek P, Masuda-Sasa T, Pokharel S, Campbell JL, et al. DNA end resection by Dna2-Sgs1-RPA and its stimulation by Top3-Rmi1 and Mre11-Rad50-Xrs2. Nature. 2010;467(7311):112–6. doi: 10.1038/nature09355 ; PubMed Central PMCID: PMC3089589. 20811461 PMC3089589

[58] Rossi SE, Foiani M, Giannattasio M. Dna2 processes behind the fork long ssDNA flaps generated by Pif1 and replication-dependent strand displacement. Nat Commun. 2018;9(1):4830. Epub 20181116. doi: 10.1038/s41467-018-07378-5 ; PubMed Central PMCID: PMC6240037. 30446656 PMC6240037

[59] Formosa T, Nittis T. Dna2 mutants reveal interactions with Dna polymerase alpha and Ctf4, a Pol alpha accessory factor, and show that full Dna2 helicase activity is not essential for growth. Genetics. 1999;151(4):1459–70. doi: 10.1093/genetics/151.4.1459 ; PubMed Central PMCID: PMC1460564. 10101169 PMC1460564

[60] Nishimura K, Fukagawa T, Takisawa H, Kakimoto T, Kanemaki M. An auxin-based degron system for the rapid depletion of proteins in nonplant cells. Nat Methods. 2009;6(12):917–22. Epub 20091115. doi: 10.1038/nmeth.1401 . 19915560

[61] Hayashi K. The interaction and integration of auxin signaling components. Plant Cell Physiol. 2012;53(6):965–75. Epub 20120319. doi: 10.1093/pcp/pcs035 . 22433459

[62] Balasubramanian S, Andreani M, Andrade JG, Saha T, Sundaravinayagam D, Garzon J, et al. Protection of nascent DNA at stalled replication forks is mediated by phosphorylation of RIF1 intrinsically disordered region. Elife. 2022;11. Epub 20220413. doi: 10.7554/eLife.75047 ; PubMed Central PMCID: PMC9007588. 35416772 PMC9007588

[63] Thakar T, Moldovan GL. The emerging determinants of replication fork stability. Nucleic Acids Res. 2021;49(13):7224–38. Epub 2021/05/13. doi: 10.1093/nar/gkab344 ; PubMed Central PMCID: PMC8287955. 33978751 PMC8287955

[64] Kaliraman V, Brill SJ. Role of SGS1 and SLX4 in maintaining rDNA structure in Saccharomyces cerevisiae. Curr Genet. 2002;41(6):389–400. Epub 2002/09/14. doi: 10.1007/s00294-002-0319-6 ; PubMed Central PMCID: PMC2804045. 12228808 PMC2804045

[65] Chakhparonian M, Faucher D, Wellinger RJ. A mutation in yeast Tel1p that causes differential effects on the DNA damage checkpoint and telomere maintenance. Curr Genet. 2005;48(5):310–22. Epub 20051104. doi: 10.1007/s00294-005-0020-7 . 16228207

[66] Lebdy R, Patouillard J, Larroque M, Urbach S, Abou Merhi R, Larroque C, et al. The organizer of chromatin topology RIF1 ensures cellular resilience to DNA replication stress. Life Sci Alliance. 2023;6(4). Epub 20230206. doi: 10.26508/lsa.202101186 ; PubMed Central PMCID: PMC9906048. 36746532 PMC9906048

[67] Munden A, Rong Z, Sun A, Gangula R, Mallal S, Nordman JT. Rif1 inhibits replication fork progression and controls DNA copy number in Drosophila. Elife. 2018;7. Epub 20181002. doi: 10.7554/eLife.39140 ; PubMed Central PMCID: PMC6185109. 30277458 PMC6185109

[68] Nasa I, Rusin SF, Kettenbach AN, Moorhead GB. Aurora B opposes PP1 function in mitosis by phosphorylating the conserved PP1-binding RVxF motif in PP1 regulatory proteins. Sci Signal. 2018;11(530). Epub 2018/05/17. doi: 10.1126/scisignal.aai8669 ; PubMed Central PMCID: PMC6454879. 29764992 PMC6454879

[69] Lu H, Davis AJ. Human RecQ Helicases in DNA Double-Strand Break Repair. Front Cell Dev Biol. 2021;9:640755. Epub 2021/03/16. doi: 10.3389/fcell.2021.640755 ; PubMed Central PMCID: PMC7947261. 33718381 PMC7947261

[70] Hegnauer AM, Hustedt N, Shimada K, Pike BL, Vogel M, Amsler P, et al. An N-terminal acidic region of Sgs1 interacts with Rpa70 and recruits Rad53 kinase to stalled forks. EMBO J. 2012;31(18):3768–83. Epub 20120720. doi: 10.1038/emboj.2012.195 ; PubMed Central PMCID: PMC3442269. 22820947 PMC3442269

[71] Chen X, Niu H, Chung WH, Zhu Z, Papusha A, Shim EY, et al. Cell cycle regulation of DNA double-strand break end resection by Cdk1-dependent Dna2 phosphorylation. Nat Struct Mol Biol. 2011;18(9):1015–9. Epub 20110814. doi: 10.1038/nsmb.2105 ; PubMed Central PMCID: PMC3168961. 21841787 PMC3168961

[72] Takahashi YH, Westfield GH, Oleskie AN, Trievel RC, Shilatifard A, Skiniotis G. Structural analysis of the core COMPASS family of histone H3K4 methylases from yeast to human. Proc Natl Acad Sci U S A. 2011;108(51):20526–31. Epub 20111207. doi: 10.1073/pnas.1109360108 ; PubMed Central PMCID: PMC3251153. 22158900 PMC3251153

[73] Gali VK, Dickerson D, Katou Y, Fujiki K, Shirahige K, Owen-Hughes T, et al. Identification of Elg1 interaction partners and effects on post-replication chromatin re-formation. PLoS Genet. 2018;14(11):e1007783. Epub 20181112. doi: 10.1371/journal.pgen.1007783 ; PubMed Central PMCID: PMC6258251. 30418970 PMC6258251

[74] Laughery MF, Hunter T, Brown A, Hoopes J, Ostbye T, Shumaker T, et al. New vectors for simple and streamlined CRISPR-Cas9 genome editing in Saccharomyces cerevisiae. Yeast. 2015;32(12):711–20. Epub 20150921. doi: 10.1002/yea.3098 ; PubMed Central PMCID: PMC4715497. 26305040 PMC4715497

[75] Piatti S, Bohm T, Cocker JH, Diffley JF, Nasmyth K. Activation of S-phase-promoting CDKs in late G1 defines a "point of no return" after which Cdc6 synthesis cannot promote DNA replication in yeast. Genes Dev. 1996;10(12):1516–31. Epub 1996/06/15. doi: 10.1101/gad.10.12.1516 . 8666235

[76] Kushnirov VV. Rapid and reliable protein extraction from yeast. Yeast. 2000;16(9):857–60. doi: 10.1002/1097-0061(20000630)16:9&lt;857::AID-YEA561&gt;3.0.CO;2-B . 10861908

[77] Katou Y, Kaneshiro K, Aburatani H, Shirahige K. Genomic approach for the understanding of dynamic aspect of chromosome behavior. Methods Enzymol. 2006;409:389–410. doi: 10.1016/S0076-6879(05)09023-3 . 16793414

