# Supplementary figures and images for: Checkpoint phosphorylation sites on budding yeast Rif1 protect nascent DNA from degradation by Sgs1-Dna2
Source: PLoS Genet. 2023 Nov 13;19(11):e1011044. doi: 10.1371/journal.pgen.1011044 (PMC10681312; doi:10.1371/journal.pgen.1011044)

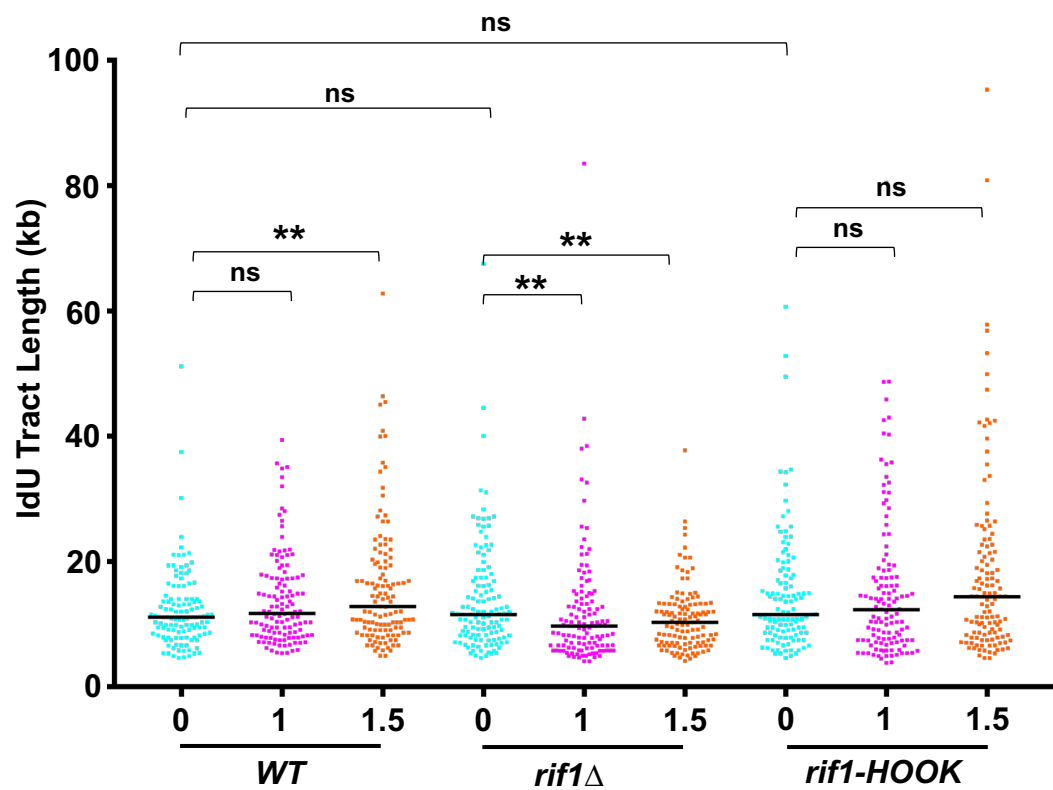

Figure S1

Supplement: S1 Fig — Rif1 HOOK domain mutant, defective in DNA binding as described in [8], is not defective in nascent DNA protection. Black horizontal bars indicate median values. ** indicates p-values less than 0.01, obtained by Mann-Whitney-Wilcoxon test. ns means “not significant”. Strains used were VGY86 (WT), CMY6 (rif1Δ), VGY318 (rif1-HOOK). (PDF) [file pgen.1011044.s001.pdf]

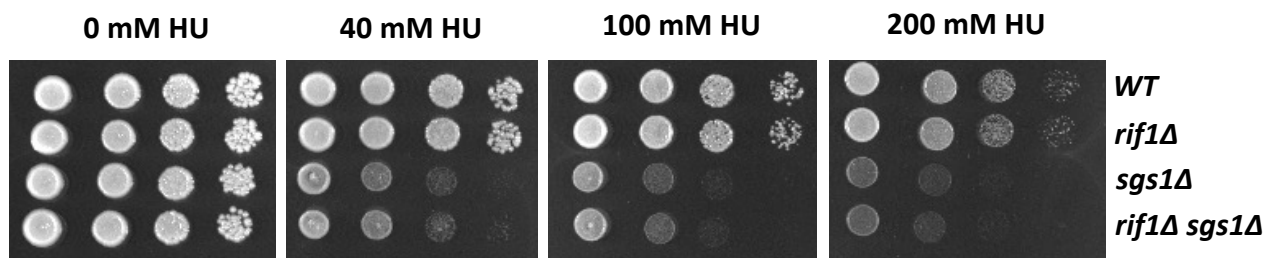

Figure S2

Supplement: S2 Fig — Ten-fold serial dilutions of indicated strains were plated on YPD with Hydroxyurea then incubated at 30°C for 2–3 days. Strains were VGY85 (WT), CMY6 (rif1Δ), CMY52 (sgs1Δ), CMY53 (sgs1Δ rif1Δ). (PDF) [file pgen.1011044.s002.pdf]

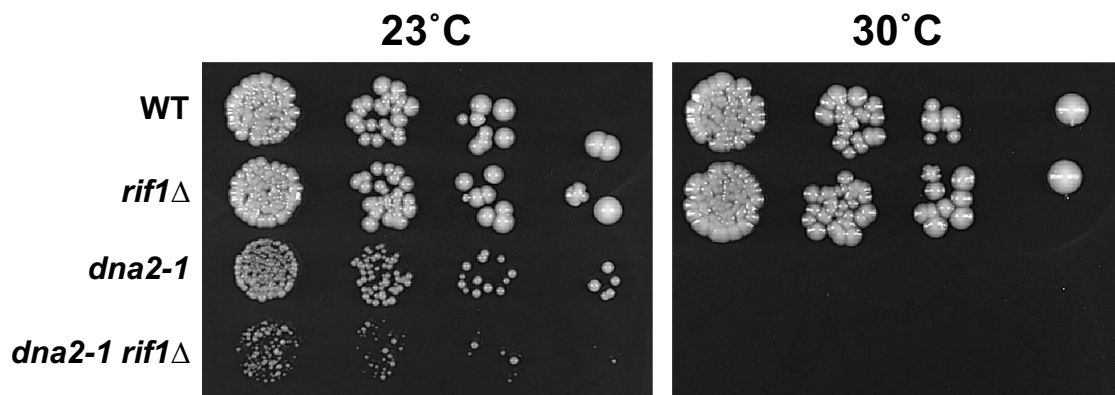

Figure S3

Supplement: S3 Fig — Serial dilutions (1:5) of cells grown on YPD at 23°C and 30°C. dna2-1 mutants are temperature sensitive and fail to grow above 30°C. Plates were imaged after 4 and 3 days respectively. (PDF) [file pgen.1011044.s003.pdf]

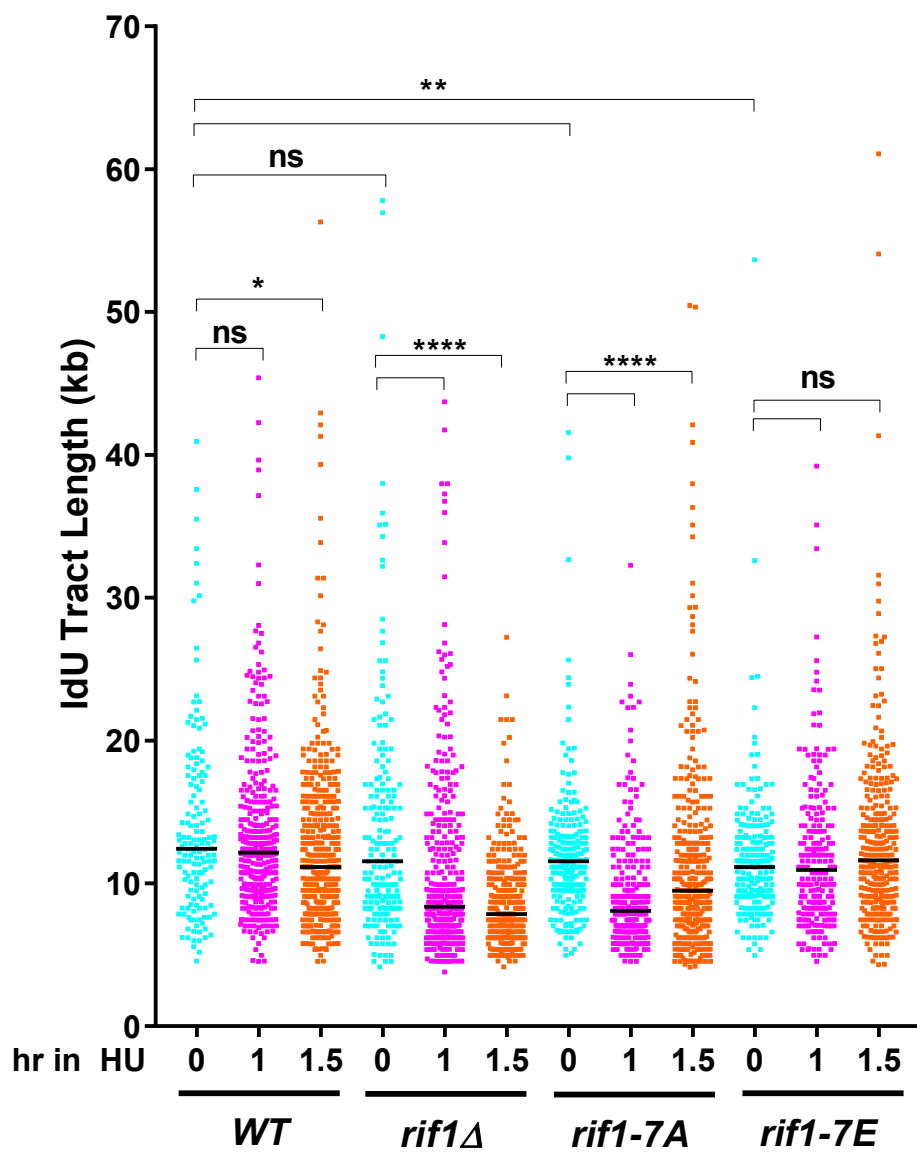

Figure S4

Supplement: S4 Fig — Analysis of the Rif1 S/TQ phospho-dead mutant rif1-7A reveals a defect in the protection of nascent DNA during an HU block, while fork protection is intact in the phosphomimic mutant rif1-7E. Black horizontal bars indicate median values. *, ** and **** indicates p-values less than 0.05, 0.01 and 0.0001, respectively, obtained by Mann-Whitney-Wilcoxon test. ns means “not significant”. (PDF) [file pgen.1011044.s004.pdf]

A

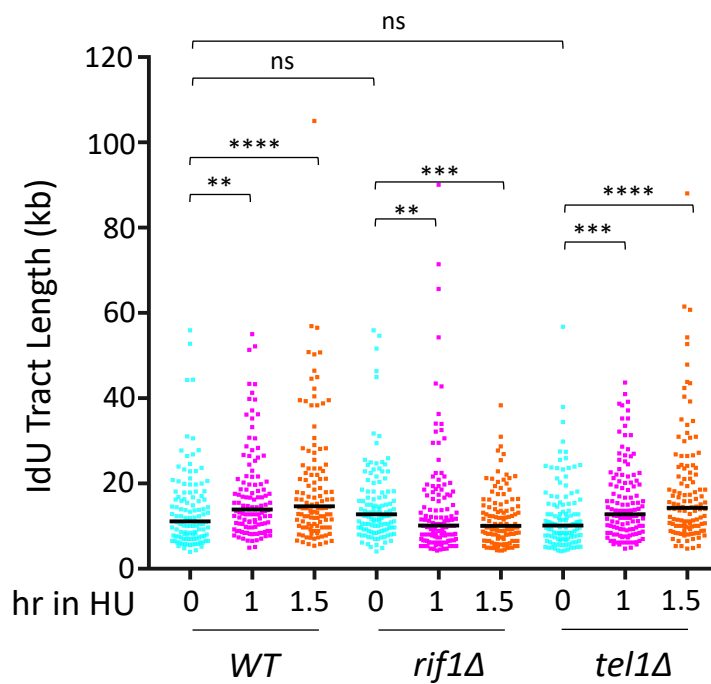

B

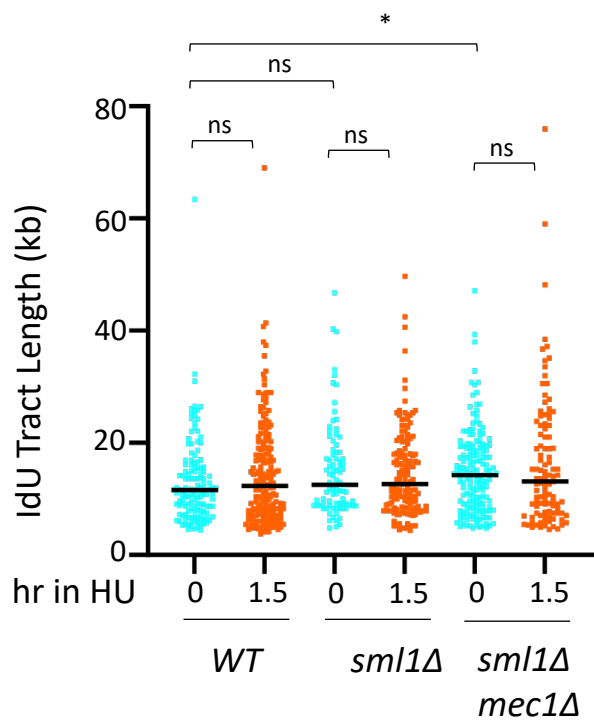

Figure S5

Supplement: S5 Fig — Black horizontal bars indicate median values. *, ** and **** indicates p-values less than 0.05, 0.01 and 0.0001, respectively, obtained by Mann-Whitney-Wilcoxon test. ns means “not significant”. Strains were (A) VGY85 (WT), CMY6 (rif1Δ), and VGY313 (tel1Δ); and (B) VGY85 (WT), CMY140 (sml1Δ), and CMY152 (sml1Δ mec1Δ). (PDF) [file pgen.1011044.s005.pdf]

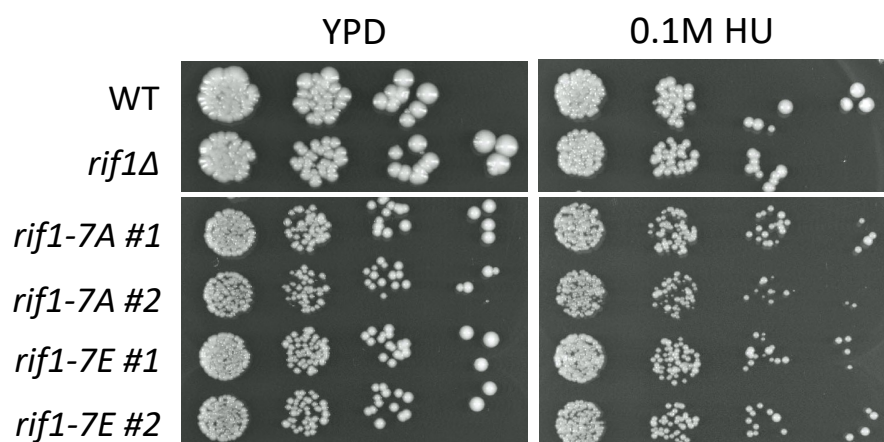

Figure S6

Supplement: S6 Fig — Serial dilutions of indicated strains were plated on YPD with no drug or with 0.1 M Hydroxyurea, then incubated at 30°C. Strains were VGY85 (WT), CMY6 (rif1Δ), duplicate strain isolates CMY130 & CMY131 (rif1-7A) and duplicate strain isolates CMY 132 & CMY133 (rif1-7E). (PDF) [file pgen.1011044.s006.pdf]

**A****Rif1-9V5 ChIP**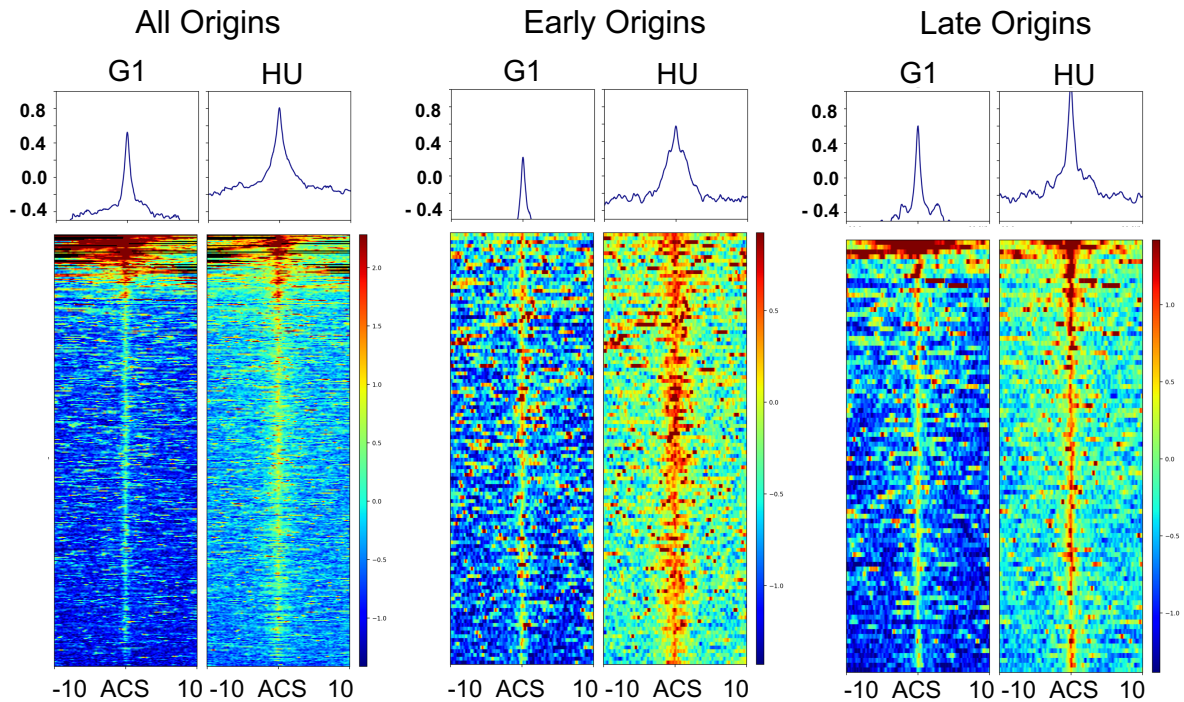**B**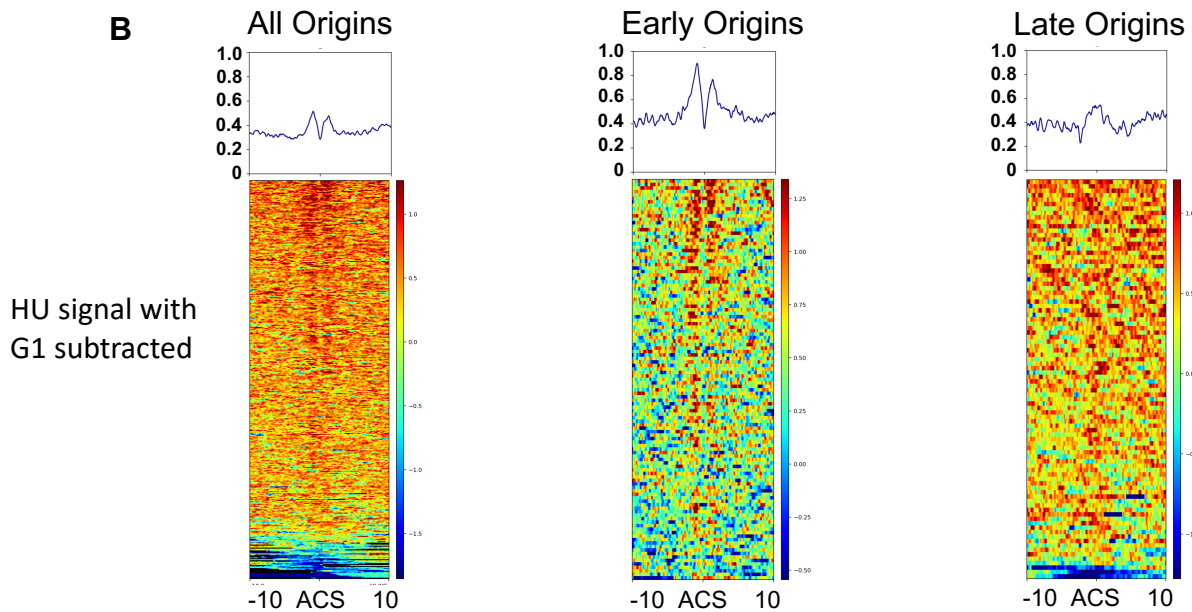

Figure S7

Supplement: S7 Fig — (A) ChIP-Seq experiment data showing enrichment of Rif1-9V5 represented as heatmaps of signal at all replication origins (left), early-initiating origins (centre) or late-initiating replication origins (right; telomere-proximal late origins excluded). Heatmaps are shown for α factor-arrested (G1) and for HU-blocked cultures. In G1-arrested cells, Rif1 binds to both early and late origins, as previously described [42]. In HU-arrested cells, broadened distribution of Rif1-9V5 signal around early (but not late) origins is indicative of replication forks diverged from early (but not late) origins. Cells from Rif1-9V5 tagged strains were collected in either G1 phase (alpha-factor arrest) or after release into S phase in the presence of 0.2M HU (23°C, 75 min). Formaldehyde-crosslinked cells were used for IP with anti-V5 antibody. IP values normalised against Input samples were used to generate heatmaps (A) at all origins of replication (410 regions) or early origins (115 regions) or late origins (90 regions) as listed in S4 Table. (B) Heatmaps showing signal observed in HU-blocked cultures after subtraction of signal observed in G1 phase. (PDF) [file pgen.1011044.s007.pdf]

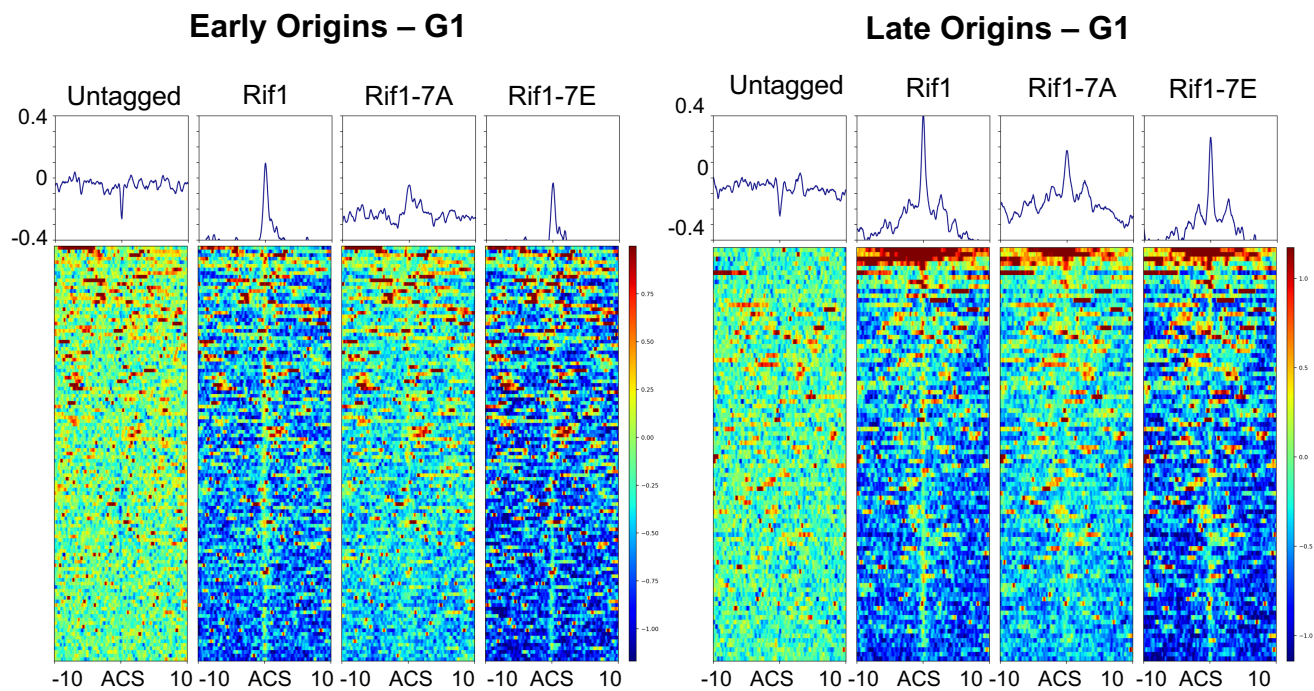

Figure S8

Supplement: S8 Fig — ChIP-Seq experiment data showing enrichment of Rif1-9V5 represented as heatmaps of signal at early replication origins (left; 115 regions) or late replication origins (right; 90 regions, telomere-proximal late origins excluded). Strains were arrested in G1 phase before collection for ChIP-Seq analysis. Data for the same experiment for cells blocked by 0.2M Hydroxurea (23°C for 75 mins) is shown in Fig 5. (PDF) [file pgen.1011044.s008.pdf]

# Rif1-13Myc ChIP

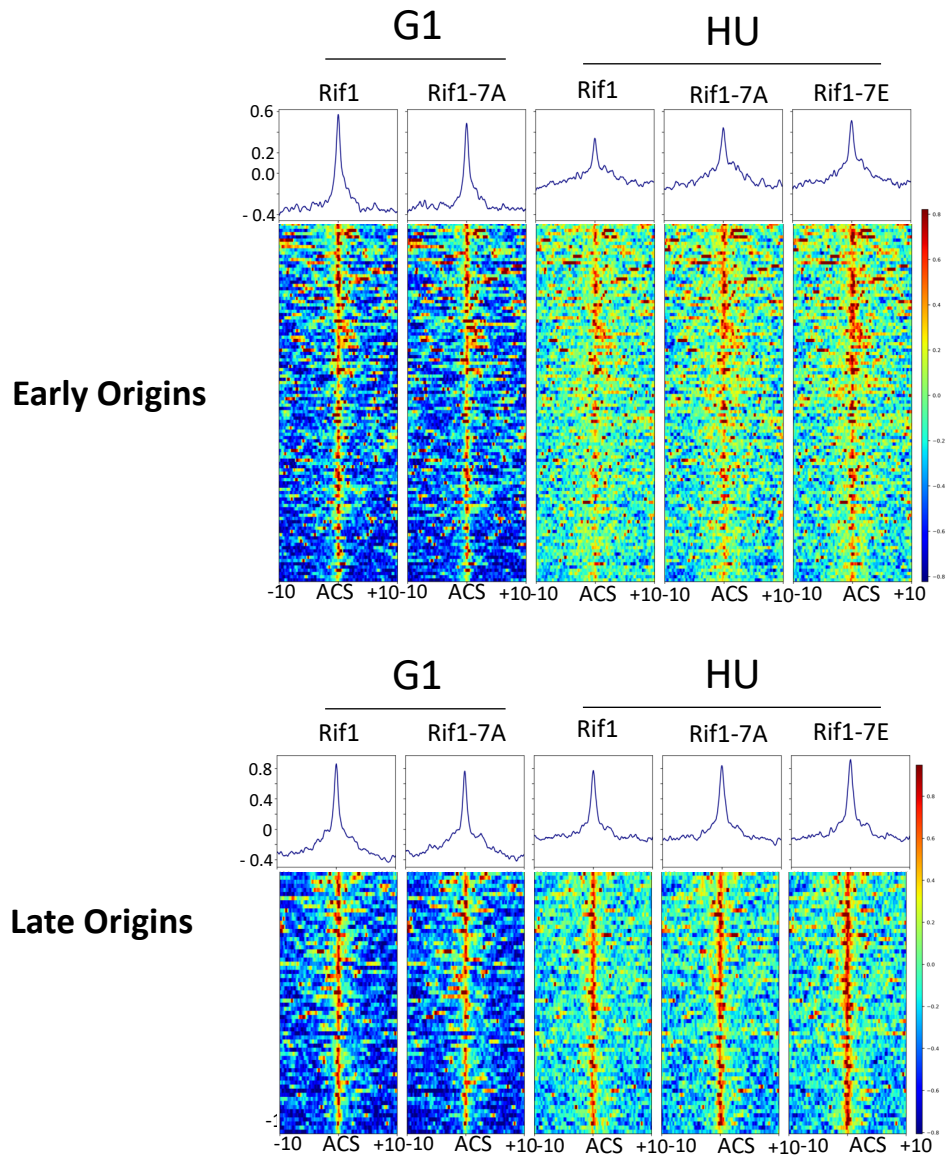

Figure S9

Supplement: S9 Fig — Data from ChIP-Seq experiment using Rif1-13Myc tag, confirming analysis in Fig 5 showing that Rif1-7A mutant is not defective in recruitment to blocked replication forks. Experimental procedure was the same as that described for Figs 5 and, but using Rif1-13Myc instead of Rif1-9V5. Strains were YSM20 (RIF1), VGY333 (rif1-7A), VGY335 (rif1-7E). (PDF) [file pgen.1011044.s009.pdf]
